# Global Vegetable Intake and Supply Compared to Recommendations: A Systematic Review

**DOI:** 10.3390/nu12061558

**Published:** 2020-05-27

**Authors:** Aliki Kalmpourtzidou, Ans Eilander, Elise F. Talsma

**Affiliations:** 1 Division of Human Nutrition and Health, Wageningen University, PO Box 17, 6700 AA Wageningen, Gelderland, The Netherlands; alikikalb@hotmail.com; 2 Unilever Foods Innovation Centre, Bronland 14, 6708 WH Wageningen, Gelderland, The Netherlands; ans.eilander@unilever.com

**Keywords:** vegetables, vegetable consumption, dietary intake, vegetable supply, global

## Abstract

Low vegetable intake is associated with higher incidence of noncommunicable diseases. Data on global vegetable intake excluding legumes and potatoes is currently lacking. A systematic review following Preferred Reporting Items for Systematic Reviews and Meta-Analyses (PRISMA) guidelines was conducted to assess vegetable consumption and supply in adult populations and to compare these data to the existing recommendations (≥240 g/day according to World Health Organization). For vegetable intake data online, websites of government institutions and health authorities, European Food Safety Authority (EFSA) Comprehensive European Food Consumption Database, STEPwise approach to surveillance (STEPS) and Pubmed/Medline databases were searched from March 2018 to June 2019. Vegetable supply data was extracted from Food Balance Sheets, Food and Agriculture Organization Corporate Statistical Database (FAOSTAT), 2013. Vegetable intake was expressed as means and 95% confidence intervals. Data were summarized for each region by calculating weighted means. Vegetable intake and supply data were available for 162 and 136 countries, respectively. Weighted mean vegetable intake was 186 g/day (56–349 g/day). Weighted mean vegetable supply was 431 g/day (71–882 g/day). For 88% of the countries vegetable intake was below the recommendations. Public health campaigns are required to encourage vegetable consumption worldwide. In the 61% of the countries where vegetable supply is currently insufficient to meet the recommendations, innovative food system approaches to improve yields and decrease post-harvest losses are imperative.

## 1. Introduction

The incidence of chronic, noncommunicable diseases (NCDs) (mainly cardiovascular diseases (CVDs), cancer, chronic respiratory disease and diabetes) is increasing rapidly and is having a significant impact on society, economy and health [1,2,3,4]. Currently, 70% of deaths are caused by NCDs worldwide [1]. A major risk factor for NCDs is an unhealthy diet, including low vegetable consumption [4,5].

Multiple studies indicated a positive relation between vegetable intake and reduction of CVDs [3,6,7,8,9,10,11,12,13,14,15,16,17,18]. In a meta-analysis by Zhan et al. (2017) [18], the pooled relative risk (RR) for CVDs between highest versus lowest vegetable intake category was 0.87 (95% CI: 0.83–0.91), which indicates a decreased CVD risk with increasing vegetable intake. Associations between vegetable intake and adiposity [2,19,20,21], type 2 diabetes [2,22,23,24,25,26,27,28,29], chronic respiratory diseases [30,31] and cancer [2,8,32,33,34,35,36,37,38] are less consistent. The benefits of vegetables on prevention of NCDs can be explained by the relatively high content of micronutrients, antioxidant compounds, polyphenols and fibers in vegetables that may counteract the biochemical processes leading to onset of CVDs and other NCDs [39]. As different vegetable categories differ in nutritional composition, the associations with health outcomes may differ as well [39].

Although the health benefits of fruits and vegetables are usually studied together in most studies [1,11,13,15,16,23,25,32,34], it is recommended that the benefits of these two food groups are assessed separately. Fruits and vegetables share health benefits due to common phytochemicals (e.g., phenolics, flavonoids, carotenoids), vitamins (e.g., vitamin C, folate, pro-vitamin A), minerals (e.g., potassium, calcium, magnesium) and fibers, but bioactive compounds differ widely in composition and ratio between fruits and vegetables [40,41,42,43]. Moreover, fruits have usually a higher concentration of sugars than vegetables, while vegetables are more likely to have a higher concentration of fibers and proteins [40]. Particularly when fruit juices are included in fruit measurements, beneficial effects of fruits alone or in combination with vegetables may not become apparent due to higher intakes in sugar and energy from fruit juices [44]. In fact, studies that have separated fruit and vegetable groups have shown differences in health outcomes [6,22,24,36,38,40,45,46,47]. For instance, in a study by Villegas et al. (2008), the increased intake of specific vegetable groups was significantly inversely associated with the type 2 diabetes risk, while this association was not found for increased fruit intake in Chinese women [24].

According to World Health Organization (WHO) and Food and Agriculture Organization (FAO) guidelines (2003), the recommended consumption of fruits and vegetables is at least 400 g/day [48]. While it is generally believed that vegetable intake is inadequate in most countries [49,50,51,52], there is currently no systematic review that focuses solely on vegetable intake to confirm these assumptions. The Global Burden of Disease (GBD) Study 2010 assessed vegetable intake and concluded that the intake was generally too low to minimize the risk of chronic diseases [49,50]. However, in this study vegetable intake was combined with that of legumes and not all data included was assessed at the individual level. Hall et al. (2009) [52] concluded that low vegetable and fruit consumption is higher in low- and middle-income countries, but did not separate fruit and vegetable intake. 

Therefore, the main aim of the current study is to systematically review the vegetable consumption in adult populations of 18 years and older globally and to compare these data to the existing recommendations and to vegetable supply data.

## 2. Materials and Methods 

### 2.1. Definitions

The definition of “vegetables” varies significantly among different countries and regions and consequently local recommendations on vegetable intake differ. In the current review, the definition for “vegetables” by WHO/FAO is followed, which excludes potatoes, tubers, legumes and pulses [53].

We derived a vegetable intake recommendation from the global recommendation by FAO/WHO (2003) of ≥400 g/day (5 servings of 80 g) for fruit and vegetables, as there is no specific guideline for vegetables alone [48]. As most countries follow the FAO/WHO guidelines and the majority suggest that at least three servings (240g/day) should come from vegetables [54,55], we set the vegetable intake recommendation at ≥240 g/day. 

### 2.2. Search Strategy

The search was conducted according to the Preferred Reporting Items for Systematic Reviews and Meta-Analyses (PRISMA) guidelines from March 2018 to June 2019 (see Supplementary Materials, Table S1) [56]. Data about vegetable intake was preferably taken from national nutrition surveys in order to extract the most representative vegetable intake data. Therefore, we first searched these surveys per country from government websites and online databases from health authorities [57,58]. The European Food Safety Authority (EFSA) Comprehensive European Food Consumption Database was used as a guide for reviewing the existing national nutrition surveys in Europe [59]. For some European countries for which the national survey reports did not report vegetable intake separately from potatoes and legumes, vegetable intake data was taken from the EFSA database. The STEPwise approach to surveillance (STEPS) database was also consulted for available vegetable intake data. STEPS is a simple, standardized method for collecting, analyzing and disseminating data regarding surveillance of NCD risk factors, with a non-itemized Food Frequency Questionnaire (FFQ) on vegetable intake. STEPS surveys are usually initiated and conducted by Ministry of Health officials in collaboration with local technical partners [4].

When for a country no nationally representative survey with relevant vegetable intake data was retrieved, a literature search in PubMed was conducted. The search string was a combination of the following terms: “dietary intake” OR “food consumption” OR “vegetable intake” OR “vegetable consumption” AND “country name”. There were no language restrictions in the overall search. Papers were screened and selected according to titles and abstracts, followed by a full-text review for final inclusion, using the following inclusion criteria:

1. Data from adult populations aged ≥18 years.
2. Free living, healthy population. Disabled people, populations with specific diseases or with special dietary needs (pregnant and lactating women, athletes) were excluded.
3. National or population-based surveys/studies. In case these were not available, baseline data or data from a control group, belonging to a healthy general population, of controlled trials were included.
4. Surveys/Studies conducted from the year 2000 onwards and published until June 2019.
5. Sample size of participants ≥100.
6. Surveys/Studies in which vegetable intake in adults at individual level were included (therefore, studies estimating vegetable intake per capita or at household level were excluded)
7. Countries and continent regions included were derived from the UN Geoscheme created by UN Statistics Division and World Atlas [57,58].

### 2.3. Data Extraction

Data extraction was conducted by two independent researchers to eliminate data extraction errors. Means and 95% confidence intervals (95% CI) were mainly extracted; if not available, medians, Standard Deviations (SD), percentiles, ranges, and Standard Errors (SE) were extracted. Additionally, data about gender, age, sample size, year of survey conduct, year of publication, study type, sample representativeness and vegetable intake assessment methodology were extracted.

### 2.4. Quality Assessment of the Data

Representativeness was defined in three levels. A sample was considered highly representative when: 1) there was a statement by the authors of the survey/study that the sample was representative for the target population, but also covered all or most of the regions of a country; 2) its representativeness was addressed by the methodology used and the stratification of the sample (distribution of sample according to age, gender, education, regions etc.). A sample was considered moderately representative when at least one of the above criteria was followed, but participants from most of the country’s regions were not included. A sample was not considered representative when none of the representativeness criteria were met [60].

### 2.5. Data Analysis

Vegetable consumption was expressed in grams per day. Descriptive statistics were used for the analysis of vegetable intake. For six countries for which no data on vegetable consumption alone were found, we used data where vegetable and fruit intake were reported together and assumed that vegetable consumption covered 60% of the intake according to WHO/FAO recommendation about vegetable and fruit intake. When means and 95% CIs were expressed for subgroups of gender, age or ethnicity, a weighted mean and 95% CI were calculated using the mean, 95% CI and the number of participants of each group. When data were not expressed in means and 95% CIs, then data were converted using the following equations.

1. For the conversion of medians and ranges into means [61]:

$$\bar{x}\;=\;(a\;+\;2m\;+\;b)/4$$

where $\bar{x}$ = mean, a = minimum, b = maximum and m = median.

2. For conversion of median and 25th and 75th percentiles into means [62]:

$$\bar{x}\;=\;(q1\;+\;m\;+\;q3)/3$$

where $\bar{x}$ = mean, q1 = P25, m = median, q3 = P75.

3. For mean and SE into 95% CIs [63]:

$$95\%CI\;=\;\bar{x}\;\pm \;1.96*SE$$

4. For mean and SD into 95% CIs [63]:

$$95\%CI\;=\;\bar{x}\;\pm \;1.96*SD/\sqrt{n}$$

5. For 25th and 75th percentiles into 95% CI [62], first a conversion was made into SD and then into 95% CI following the equation above (4):

SD = (P75−P25)/1.35

6. For 5th and 95th into 95% CI [62], first a conversion was made into SD and then into 95% CI following the equation above (4):

SD = (P95−P5)/3.3

For each region, we calculated a weighted mean vegetable intake where we used the adult population of 18 years and older in 2013 as derived from United Nations Children’s Fund (UNICEF) State of the World’s Children report, 2015 [64].

### 2.6. Vegetable Supply Data

Vegetable supply per country was extracted and calculated according to data from the most recent food balance sheets by Food and Agriculture Organization Corporate Statistical Database (FAOSTAT) (2013) [65]. The vegetable supply was the sum of the supply data from the food categories onions, tomatoes and their products, peas and other vegetables: and excluded legumes, potatoes, tubers and pulses [65]. Vegetable supply was converted from kg/year/capita to g/day/capita in order to compare results with the vegetable intake data. For each region, we calculated a weighted mean vegetable supply where we used the adult population in 2013 as weighing factor.

## 3. Results

### 3.1. Data Availability, Representativeness and Dietary Assessment Methodology

**Figure 1:**
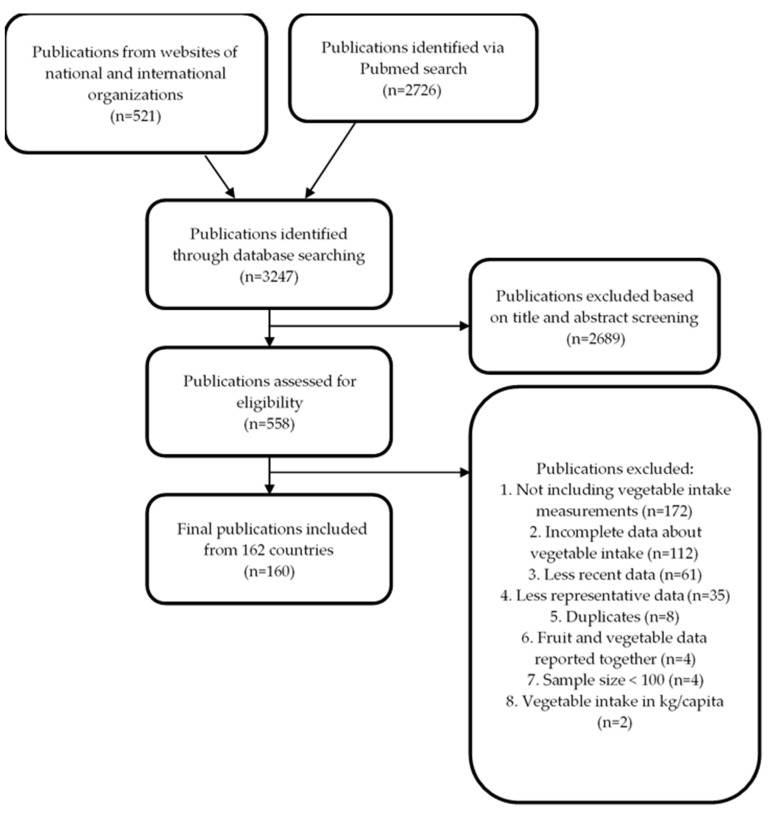
Flowchart of the review process for the selection of the final surveys/studies assessed.

The systematic search yielded initially 3247 publications (Figure 1). After abstract and title screening, 558 publications were assessed for eligibility. The final review dataset included 160 publications with studies measuring vegetable intake in 162 out of 235 countries, covering 93% of the world’s adult population. Vegetable supply data were available for 136 out of the 162 (84%) countries with vegetable intake data; for the smaller countries vegetable supply data was mostly lacking.

The surveys/studies included in this review had a high sample representativeness, with only 28 (17%) with a non-representative sample and six (4%) with no information about sample representativeness (see Supplementary Materials, Table S2). The sample size of the surveys/studies ranged from 100 subjects in Jamaica to 140,859 in China.

The most frequently used method to assess vegetable intake was a (semi-) quantitative FFQ (65%), which also includes the STEPS methodology which was used in most African, Latin American and Oceanian countries. Vegetable intake data in high-income countries were mostly from national nutrition surveys in which a combination of 24 h dietary recalls and FFQs were used (see Supplementary Materials, Table S2).

### 3.2. Global Vegetable Intake and Supply

Based on data of 162 countries, the weighted mean vegetable intake was 186 g/day and ranged from 56 g/day in Central America to 349 g/day in East Asia (Table 1). Based on data from 136 countries, the weighted mean global vegetable supply was 431 g/day and ranged widely among the different regions from 71 g/day in Melanesia to 882 g/day in East Asia. Details of vegetable intake and supply per country can be found in the supporting information, Table S2. Asia was the continent with the highest vegetable consumption, where 10 countries (29%) met the recommendations, while in Oceania 2 (11%), Africa 3 (7%), Europe 4 (11%) and America 1 (7%) countries had an adequate consumption of vegetables (Table 1). In Europe, 26 out of 36 (72%) and in Asia, 19 out of 31 countries (61%) had an adequate vegetable supply to meet the vegetable recommendation of ≥240 g/day; whereas in Africa 5 out of 39 countries (13%) had a sufficient vegetable supply to meet the recommendation.

**Figure 2:**
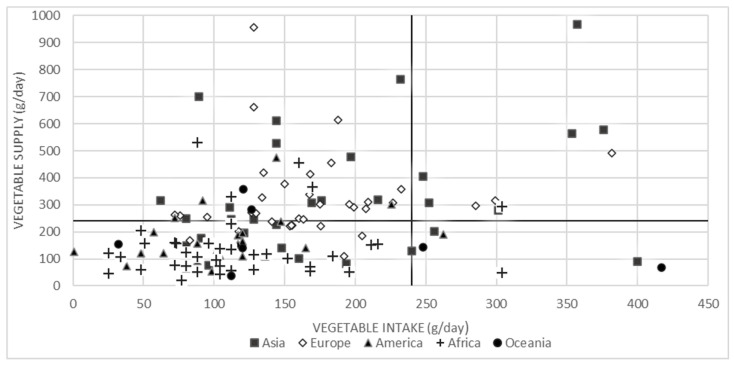
Vegetable intake and supply per country benchmarked against the vegetable recommendations of 240 g/day (WHO/FAO).

Figure 2 provides an overview of the vegetable intake and supply data against the vegetable recommendations of ≥240 g/day based on data of 136 countries. Only 10 out of 136 countries (7%) have an adequate intake and adequate supply of vegetables, whereas in 119 of the 136 countries (88%) vegetable intake is below the recommendations. Moreover, in 73 of these 119 countries (61%) vegetable supply is inadequate to meet the recommendations. For seven countries, vegetable intake was adequate, but vegetable supply data were below 240 g/day.

### 3.3. Asia

Vegetable intake data was absent for Central Asia. Among the other Asian regions, weighted mean vegetable intake ranged from 81 g/day (mean lowest and highest intake in region countries: 56, 304 g/day) in South Asia to 349 (80, 357) g/day in East Asia. In East Asia four out of six countries (67%) met the recommendations for vegetable intake, whereas this was the case for 9–29% of the countries in the other Asian regions (Table 1). The weighted mean vegetable supply ranged widely within and among the Asian regions; from 190 (92, 577) g/day in South East Asia to 882 (149, 969) g/day in East Asia. Only in South East Asia the weighted mean vegetable supply was below 240 g/day, whereas that of South Asia was just above the recommendations with 264 g/day.

### 3.4. Europe

Vegetable intake data was available for 37 out of 56 European countries and in four North European countries (Iceland, Denmark, Norway, Lithuania) legumes were included in the vegetable intake measurements. Weighted mean vegetable intake ranged from 123 (95, 207) g/day in Western Europe to 270 (83, 382) in Eastern Europe (Table 1). Out of 37 European countries, four countries met the vegetable intake recommendations of ≥240 g/day, including one in Southern and three countries in Eastern Europe. The weighted mean vegetable supply ranged from 269 (239, 379) g/day in Western Europe to 453 (221, 662) g/day in Southern Europe. In 10 out of 36 countries, the vegetable supply was not sufficient to meet the recommended intake of 240 g/day.

### 3.5. America

Vegetable intake data was available for 28 out of 54 countries in the Americas. Weighted mean vegetable intake ranged from 56 (1–88) g/day in Central America to 156 (48, 263) g/day in North America (Table 1). Of all the American countries, one South American country met the recommendations for vegetable intake. The weighted mean vegetable supply ranged from 102 (53, 252) g/day in the Caribbean to 277 (153, 474) g/day in North America. In Central and South America and the Caribbean the weighted mean vegetable supply was below 240 g/day, whereas in three out of four North American countries the vegetable supply was just above the recommendations with 277 g/day.

### 3.6. Oceania

Vegetable intake data was available for 18 out of 32 countries in Oceania. Weighted mean vegetable intake ranged from 73 (64, 248) g/day in Melanesia to 196 (72, 417) g/day in Polynesia (Table 1). The vegetable intake met the recommendation in 2 of the 18 countries. The weighted mean vegetable supply ranged from 71 (37, 142) g/day in Melanesia to 295 (283, 357) g/day in Australasia. Only in Australasian countries the vegetable supply was above 240 g/day.

### 3.7. Africa

Vegetable intake data was available for 44 out of 62 countries in Africa. The weighted mean vegetable intake ranged from 98 (28, 304) g/day in East African countries to 135 (88, 168) g/day in South Africa (Table 1). In 3 out of the 44 African countries vegetable intake recommendations were met. The weighted mean vegetable supply ranged from 86 (48, 206) g/day in East African countries to 434 (230, 531) g/day in North Africa. Only in North Africa was the weighted mean vegetable supply sufficient to meet the recommendation of 240 g/day.

## 4. Discussion

Weighted mean vegetable intake was 186 g/day and ranged from 56 g/day in Central America to 349 g/day in East Asia. Weighted mean vegetable supply was 431 g/day and ranged widely among the different regions from 71 g/day in Melanesia to 882 g/day in East Asia. For 88% of the countries, vegetable intake was below the recommendations and for 61% the vegetable supply was too low to meet the recommendations of 240 g/day.

To our knowledge this is the first systematic review evaluating global vegetable intake (excluding legume and potato intake) at an individual level with a comparison to vegetable recommendation and vegetable supply. For 82% of the surveys/studies that were identified, vegetable intake data was compliant with the vegetable definition of FAO/WHO [53]. For 4% of the countries where fruit and vegetable intake were reported together, we made the assumption that 60% of the intake would come from vegetables, which may have resulted in either over- or underestimation of the intake. Only the most recent surveys of each country were selected, with 65% of the surveys/studies conducted in the last decade. Another important strength of this review is the high representativeness of the vegetable intake data, since for nearly 80% of the countries included the data extracted was nationally representative.

Limitations of our review with regard to the vegetable intake data are mainly due to heterogeneity among studies, including differences in the methods of assessing vegetable intake, differences in the definition of vegetable intake and differences in the units of reporting. For dietary intake of vegetables, the most common method was a (semi-)quantitative FFQ at individual level, which was used in 65% of the identified surveys/studies. A recent review on dietary intake measurements of vegetable and fruits in Europe, indicated also that FFQs were mostly used [3]. This review indicated that FFQs may differ in a number of aspects, such as (non)-itemization of terms, inclusion of potatoes and legumes in vegetable definition, and portion size calculation. This may also explain part of the variation in vegetable consumption between studies in our review. For instance, in many of the studies included in our review it was unclear whether soups, vegetable juices, processed vegetables and mixed dishes containing vegetables were included and hence vegetable intake may be underestimated. Furthermore, we noticed heterogeneity in calculation and reporting of vegetable intake data among the surveys that were using the STEPS methodology. Lastly, due to heterogeneity in reporting units, it was not possible to convert all data to means and SDs and hence it was not possible to calculate the prevalence of inadequate vegetable intake per country and region. Therefore, we decided to report vegetable intake in means and 95% CI.

Vegetable supply data may be underestimated as the data from FAO Food Balance Sheets are reported per capita and therefore comprise data for the entire population, including children. Furthermore, exclusion of vegetables that are cropped privately or in the wild [53,66] and unavailability of production data of all regions within a country [66] in Food Balance Sheet data may have led to underestimation of vegetable supply. These limitations may partly explain the finding that for some countries the vegetable supply was lower than the intake. Other reasons for this finding can be attributed to limitations of the studies’ measuring of vegetable intake, e.g., non-representativeness of intake data, lack of addressing seasonal differences in vegetable intake, and large differences between the year that the intake data were collected and the year of the supply data.

Our finding that vegetable intake worldwide is low (186 g/day) with many low- and middle-income countries in the range of 1–2 portions per day, correspond with that of earlier reviews. The GBD study of 2010 reported a mean intake of 209 g/day including legumes and starchy vegetables [49,50]. Similarly, the Prospective Urban Rural Epidemiology (PURE) study [51] and World Health Survey 2002–2003 [52] indicated that fruit and vegetable intake were below the recommendations and the intakes were generally lower in low- and middle income countries. One of the main reasons for the low vegetable intake in the low- and middle-income countries is urbanization and shift to a more “western” diet, high in foods that are high in sugar, fat, and salt and low in fiber. In addition, in these countries there is generally a lack of secure availability and affordability of vegetables, which are underlying causes for an inadequate intake of vegetables [51].

An important finding of our review is that even though the weighted vegetable supply worldwide is sufficient (431 g/day), vegetable supply among the countries varies widely and consequently in many countries it is insufficient to adhere to the recommended intake of 240 g/day. With a shift to a more plant-based diet, as recently recommended by the EAT-Lancet commission, vegetable intake recommendations are further increasing to 300 g/day [67]. This would imply that in the vast majority (93%) of the countries globally, vegetable supply would not be sufficient. The EAT-Lancet commission predicts that an increase of 75% in production of vegetables is needed together with accompanying measures to prevent losses in vegetable supply, e.g., preservation techniques such as drying, canning and freezing of vegetables. Moreover, improvements in the infrastructure are needed to facilitate the distribution of vegetables and innovative approaches to improve accessibility of fresh vegetables to consumers are essential to improve vegetable consumption.

To further improve vegetable intake, consumers should be educated about the benefits of vegetables for health. International recommendations should become more specific concerning the categories included and the portion sizes of raw and cooked vegetables. The development of country specific food based dietary guidelines including a recommendation for vegetable intake can play a role in this, although many countries have not yet developed these or the recommendation is unclear [68]. Subsequently, the national nutrition policy makers could develop detailed guidelines which are in line with the international guidelines by WHO/FAO and translated to the dietary goals and traditions of the country. These guidelines should include practical examples of traditional cooking methods and recipes to increase vegetable intake. Moreover, community-based activities like community kitchens could be organized [69]. The objective would be to educate the populations about healthy dietary patterns according to national dietary guidelines, including the importance of vegetables and vegetable categories, and to improve cooking and purchasing skills adapted to the national and regional culture. While the effectiveness of public health interventions to increase vegetable intake through education and counseling is mostly positive, unclarity exists about whether the achievements remain in the longer term [70,71,72]. In order to change the behavior of the consumer, it is important that the environment should be encouraging and rewarding. Besides an adequate supply and accessibility, affordability of vegetables is key. The PURE study (2016) indicated that low income countries spend a higher percentage of the households’ income on vegetables compared to high income countries [51]. Governments need to play a role to ensure that vegetables become affordable to the entire population, including the lower income consumers. A modelling study estimated that, even under optimistic socioeconomic scenarios with economic growth, the future supply of vegetables will be insufficient to meet the recommendations for many countries [73]. This prediction makes even more imperative the need to tackle the global problem of food loss and waste, especially in the case of vegetables that are considered sensitive food products and whose loss can easily occur due to poor practices and degradation [74]. Nevertheless, locally grown and sourced vegetables, and inclusion of indigenous vegetables may help to ensure a fresh and sustainable vegetable supply. Furthermore, learning from public health strategies aimed at increasing vegetable intake in the countries with an adequate vegetable intake should be taken into account.

Lastly, it would be beneficial to get a better understanding of the health properties of the different vegetables and how different processing techniques will influence the effect of vegetables on health. This could lead to more detailed guidance on vegetable consumption; for instance, in the German guidelines, specific recommendations were provided for raw and cooked vegetables. In order to monitor whether populations adhere to the guidelines and to allow for country comparisons, harmonization of the methodology used to collect vegetable intake data, including standard dietary methods and measurement units, is recommended. 

## 5. Conclusions

In conclusion, our review indicated that global vegetable intake is generally below the recommendations of 240 g/day and that the vegetable supply is mostly inadequate to meet the recommendations. With the current emphasis on consuming plant-based diets, efforts are needed to improve the production, accessibility and affordability of vegetables in conjunction with innovative approaches to establish a sustained behavior change aimed at improving vegetable intake worldwide.

## Figures and Tables

**Table 1:** Vegetable intake expressed in average and range (g/d) and number (%) of countries according to vegetable servings/d per continent region [57,58].

Continent	Region	Number of countries in the Region	Vegetable intake per Region (g/day)				Vegetable Supply per Region (g/day)			
			Countries with Data (n)	Weighted Mean	Range	Countries with Vegetable Intake ≥240 g/day (%)	Countries with Data (n)	Weighted Mean	Range	% of Vegetable Supply Consumed
Asia	West	11	11	144	89–256	9	9	545	202–765	26 ^1^
	East	8	6	349	80–357	67	6	882	149–969	40 ^1^
	Southeast	13	11	153	91–400	27	10	190	92–577	80 ^4^
	South	8	7	81	62–304	29	6	264	75–315	31 ^3^
Europe	East	13	12	270	83–382	25	12	323	109–956	84 ^2^
	North	17	10	132	72–227	0	10	270	192–339	49
	South	17	9	167	128–240	11	8	453	221–662	37
	West	9	6	123	95–207	0	6	269	239–379	46
America	Central	7	2	56	1–88	0	2	145	127–156	39
	South	12	8	156	48–263	13	8	148	64–197	106 ^3^
	Caribbean	29	14	104	38–163	0	9	102	53–252	102 ^1^
	North	6	4	108	92–226	0	4	277	153–474	39
Oceania	Australasia	5	3	126	23–127	0	2	295	283–357	43
	Melanesia	7	3	73	64–248	33	2	71	37–142	103 ^1^
	Micronesia	8	5	122	32–216	0	1	155	155–155	78
	Polynesia	12	7	196	72–417	14	2	110	67–140	178 ^1^
Africa	North	11	5	122	88–170	0	4	434	230–531	28
	East	20	14	98	28–304	7	10	86	48–206	114 ^5^
	Central	9	6	103	25–304	17	6	134	21–294	77 ^4^
	West	17	14	123	25–216	7	14	122	46–331	101 ^6^
	South	5	5	135	88–168	0	5	114	54–119	118 ^4^

^1–7^ Number indicates the number of countries in the region where the vegetable supply was higher than the vegetable intake.

## Supplementary Materials

The following are available online at https://www.mdpi.com/2072-6643/12/6/1558/s1, Table S1: PRISMA checklist, Table S2: Vegetable intake, socio-demographics, sample size and representativeness, vegetable intake data source and dietary method used per country.
