# Supplementary material for: Global Vegetable Intake and Supply Compared to Recommendations: A Systematic Review
Source: Nutrients. 2020 May 27;12(6):1558. doi: 10.3390/nu12061558 (PMC7352906; doi:10.3390/nu12061558)
Supplement: Supplementary file 1 [file nutrients-12-01558-s001.zip › MS Kalmpourtzidou_Supplementary material_Nutrients.docx]

**Table S2:** Vegetable intake, sociodemographics, sample size and representativeness, vegetable intake data source and dietary method used per country

| **Region** | **Country** | **Adult population (thousands) [1]** | **Classification of countries according to income by World Bank (2018-2019) [2]** | **Data source** | **Dietary method** | **Age (y)** | **Sample size (N)** | **Vegetable intake** *(g/d)* | | **Vegetable supply** *(g/d)* | **(Vegetable intake/ vegetable supply) %** | **Representativeness** |
| --- | --- | --- | --- | --- | --- | --- | --- | --- | --- | --- | --- | --- |
|  |  |  |  |  |  |  |  | **Mean** | **95% CI** |  |  |  |
| West Asia | Iran [3] | 55527 | Upper-middle | STEPS National survey 2011 | FFQ | 15-64 | 11460 | 89 | 79-98 | 701 | 46 | Yes ^h^ |
|  | Iraq [4] | 18013 | Upper-middle | STEPS National survey 2015 | FFQ | ≥18 | 4063 | 176 | 168-184 | 316 | 56 | Yes ^h^ |
|  | Israel [5] | 5224 | High | Rav Mabat Adult- Second National Health and Nutrition Survey, Ages 18-64, 2014-2016 | 24h recall | 18-64 | 2904 | 197 | 188-206 | 479 | 52 | Yes ^h^ |
|  | Jordan [6] | 4374 | Upper-middle | STEPS National survey 2007 | FFQ | ≥18 | 3654 | 216 | - | 320 | 68 | Yes ^h^ |
|  | Kuwait [7] | 2394 | High | STEPS National survey 2014 | FFQ | 18-69 | 3883 | 144 | 144-152 | 612 | 24 | Yes ^h^ |
|  | Lebanon [8] | 3548 | Upper-middle | STEPS National survey 2016-2017 | FFQ | 18-69 | 1702 | 144 | 128-168 | 529 | 27 | Yes ^h^ |
|  | Qatar [9] | 1822 | High | STEPS National survey 2012 | FFQ | 18-64 | 2496 | 112 | 96-128 | - | - | Yes ^h^ |
|  | Saudi Arabia [10] | 19140 | High | The Saudi Health Interview Survey (SHIS) 2013 | diet history | 15-64 | 9855 | 111 | 107-115 | 290 | 35 | Yes ^h^ |
|  | United Arab Emirates [11] | 7680 | High | population-based study | 24h recall, FFQ | 18-65 | 200 | 256 | 233-279 | 202 | 127 | No |
|  | Palestine [12] | 2275 | Low | STEPS National survey 2010-2011 | FFQ | 15-64 | 6957 | 144 | 120-160 | - | - | Yes ^h^ |
|  | Uzbekistan [13] | 18939 | Lower-middle | STEPS National survey 2014 | FFQ | 18-64 | 4350 | 232 | 208-248 | 765 | 30 | Yes ^h^ |
| East Asia | China [14] | 1059973 | Upper-middle | 2013 China Chronic Disease Surveillance survey | FFQ | 18-64 | 140859 | 357 | 345-369 | 969 | 58 | Yes ^h^ |
|  | Japan [15] | 106949 | High | National cross-sectional fruit and vegetable consumption study | daily food record | 20-69 | 1830 | 301 | 294-309 | 281 | 107 | Yes ^m^ |
|  | South Korea [16,17] | 39924 | High | Korea National Health and Nutrition Survey 2007-2009 | 24h recall, FFQ | 40-64 | 5509 | 354 | - | 564 | 63 | Yes ^h^ |
|  | Mongolia [18] | 1923 | Lower-middle | STEPS National survey 2013 | FFQ | 15-64 | 6013 | 80 | 72-88 | 149 | 54 | Yes ^h^ |
|  | Hong Kong [19] | 5652 | High | Hong Kong population-based food consumption survey 2005-2007 | 2d 24h recall, FFQ | 20-84 | 5008 | 169 | 169-172 | 307 | 55 | Yes ^h^ |
|  | Taiwan [20] | 18403 | High | Nutrition and Health Survey in Taiwan (NAHSIT) 2005-2008 | 24h recall | 19-64 | 1942 | 252 ^l^ | - | 308 | 82 | Yes ^h^ |
| Southeast Asia | Brunei [21] | 292 | High | STEPS National survey 2015-2016 | FFQ | 18-69 | 3808 | 128 | 120-136 | 247 | 52 | Yes ^m^ |
|  | Indonesia [22] | 164360 | Lower-middle | STEPS National survey 2006 | FFQ | 25-64 | 1242 | 136 | 128-136 | 111 | 123 | No |
|  | Cambodia [23] | 9552 | Lower-middle | STEPS National survey 2010 | FFQ | 25-64 | 5432 | 160 | 144-168 | 102 | 156 | Yes ^h^ |
|  | Lao PDR [24] | 3911 | Lower-middle | STEPS Subnational survey 2008 | FFQ | 25-64 | 4180 | 376 | 344-408 | 577 | 61 | No |
|  | Philipinnes [25] | 58636 | Lower-middle | 7th National nutrition survey 2008 | 2d 24h recall | 20-59 | 8679 | 91 | - | 175 | 52 | Yes ^h^ |
|  | Vietnam [26] | 66602 | Lower-middle | STEPS National survey 2015 | FFQ | 18-69 | 3734 | 248 | 240-256 | 404 | 61 | Yes ^h^ |
|  | Malaysia [27] | 20291 | Upper-middle | Malaysian Adult Nutrition Survey (MANS) Vol. II 2014 | 24h recall | 18-59 | 3000 | 121 | 116-126 | 195 | 62 | Yes ^h^ |
|  | Myanmar [28] | 37163 | Lower-middle | Cross-sectional study | FFQ | 25-74 | 1486 | 144 | 128-160 | 226 | 64 | Yes ^h^ |
|  | Singapore [29] | 4323 | High | National nutrition survey | FFQ, dietary practices questionnaire (DPQ) | 18-69 | 1647 | 173 | 166-182 | - | - | Yes ^h^ |
|  | Thailand [30] | 52149 | Upper-middle | Thailand National Health Examination Survey III | FFQ | 15-69 | 29789 | 148 | 146-149 | 142 | 105 | Yes ^h^ |
|  | Timor-Leste [31] | 517 | Lower-middle | STEPS National survey 2014 | FFQ | 18-69 | 2322 | 400 | 248-560 | 92 | 437 | Yes ^m^ |
| South Asia | Bangladesh [32] | 99929 | Lower-middle | Fruit and vegetable consumption survey in Asia | FFQ | 25-64 | 8069 | 194 | - | 82 | 236 | No |
|  | Bhutan [33] | 498 | Lower-middle | STEPS National survey 2014 | FFQ | 18-69 | 2822 | 304 | 272-328 | - |  | Yes ^h^ |
|  | India [34] | 816756 | Lower-middle | National nutrition survey | 24h recall | ≥16 | 70525 | 62 | - | 315 | 20 | Yes ^h^ |
|  | Maldives [35] | 225 | Upper-middle | STEPS National survey 2011 | FFQ | 15-64 | 1780 | 80 | 72-80 | 248 | 32 | Yes ^m^ |
|  | Nepal [36] | 16271 | Low | STEPS National survey 2013 | FFQ | 15-69 | 4143 | 112 | 104-112 | 247 | 45 | Yes ^h^ |
|  | Pakistan [37] | 108289 | Lower-middle | STEPS National survey 2014-2015 | FFQ | 18-69 | 5123 | 96 | - | 75 | 74 | Yes ^h^ |
|  | Sri Lanka [38] | 14965 | Lower-middle | STEPS National survey 2014-2015 | FFQ | 18-69 | 5188 | 240 | 224-256 | 130 | 184 | Yes ^h^ |
| East Europe | Azerbaijan [39] | 6866 | Upper-middle | STEPS National survey 2011 | FFQ | 18-64 | 1670 | 183 | 171-211 | 456 | 40 | Yes ^h^ |
|  | Armenia [40] | 2254 | Upper-middle | STEPS National survey 2016-2017 | FFQ | 18-69 | 2349 | 128 | 120-136 | 956 | 13 | Yes ^h^ |
|  | Belarus [41] | 7641 | Upper-middle | STEPS National survey 2016-2017 | FFQ | 18-69 | 5760 | 168 | 144-184 | 413 | 41 | Yes ^h^ |
|  | Bulgaria [42] | 6039 | Upper-middle | National nutrition survey 2004 | Unknown | 18-64 | 691 | 205 | 193-217 | 185 | 111 | Unknown |
|  | Czech Republic [42,43] | 8827 | High | Individual food consumption - the national study SISP04 | 24h recall | 16-64 | 1751 | 117 | 113-122 | 201 | 58 | Unknown |
|  | Georgia [44] | 3419 | Lower-middle | STEPS National survey 2016 | FFQ | 18-69 | 5554 | 192 | 184-200 | 109 | 176 | Yes ^h^ |
|  | Hungary [45] | 8186 | High | National nutrition survey 2003-2004 | 3d food record | 18-64 | 1074 | 154 | 149-159 | 222 | 69 | Unknown |
|  | Moldova [46] | 2786 | Lower-middle | STEPS National survey 2013 | FFQ | 18-69 | 4738 | 160 | 152-168 | 248 | 65 | No |
|  | Poland [47] | 31257 | High | National survey 2000 | 24h recall | 0-96 | 2527 | 285 | 281-290 | 298 | 96 | Yes ^m^ |
|  | Romania [42] | 17757 | Upper-middle | National nutrition survey | 7d food record | 19-64 | 1254 | 382 | 376-287 | 491 | 78 | Yes ^h^ |
|  | Russia [48] | 116264 | Upper-middle | HAPIEE study | FFQ | 45-69 | 9360 | 299 | 297-302 | 316 | 95 | Unknown |
|  | Slovakia [42] | 4453 | High | National nutrition survey 2008 | 24h recall | 19-59 | 2761 | 83 | 78-88 | 167 | 49 | Unknown |
| North Europe | Denmark [49] | 4421 | High | National nutrition survey 2011-2013 | 7d food record | 19-75 | 3016 | 199 ^l^ | 195-203 | 290 | 69 | Yes ^h^ |
|  | Estonia [42,50] | 1046 | High | National nutrition survey 2013-2015 | FFQ, 2d 24h recalls | 18-64 | 2442 | 167 | 161-174 | 339 | 49 | Yes ^h^ |
|  | Finland [51] | 4348 | High | The National FINDIET 2012 Survey | 48h food interviews | 25-64 | 1295 | 163 | 156-169 | 246 | 61 | Yes ^h^ |
|  | Iceland [52] | 248 | High | National nutrition survey 2010-2011 | 2d 24h recalls, FFQ | 18-80 | 1312 | 120 ^l^ | 115-125 | 192 | 125 | Yes ^h^ |
|  | Ireland [53] | 3452 | High | National nutrition survey 2008-2010 | 4d food record, FFQ | 18-64 | 1274 | 76 | 74-78 | 259 | 50 | Yes ^h^ |
|  | Latvia [42,54] | 1690 | High | National nutrition survey 2014 | FFQ, dietary recall, dietary record | 18-64 | 1216 | 209 | 200-217 | 310 | 67 | Yes ^h^ |
|  | Lithuania [55] | 2455 | High | National nutrition survey 2013-2014 | 24h recall | 19-64 | 2213 | 227^l^ | 219-235 | 308 | 74 | Yes ^h^ |
|  | Norway [56] | 3906 | High | Norkost 3 | 2d 24h recalls | 18-70 | 1787 | 155 ^l^ | 150-160 | 225 | 69 | Yes ^h^ |
|  | Sweden [42,57] | 7642 | High | National nutrition survey 2010-2011 | FFQ, 4d food record | 18-64 | 1430 | 72 | 70-74 | 262 | 28 | Yes ^h^ |
|  | UK [42,58] | 49825 | High | National Diet and Nutrition Survey 2010-2011 | 2 FFQ, 4d food record | ≥19 | 1031 | 126 | 120-133 | 271 | 47 | Yes ^h^ |
| South Europe | Andorra [59] | 65 | High | National nutrition survey 2004-2005 | 2d 24h recalls, FFQ | 25-64 | 665 | 240 | 219-261 | - | - | Yes ^h^ |
|  | Croatia [42,60] | 3504 | High | National Food Consumption Survey 2011-2012 | 24h recall, FFQ | 18-64 | 2002 | 176 | 169-183 | 221 | 80 | Yes ^h^ |
|  | Greece [61] | 9178 | High | HYDRIA survey | 24h recalls, FFQ | ≥18 | 4011 | 188 | 182-193 | 615 | 31 | Yes ^h^ |
|  | Italy [62] | 50682 | High | INRAN-SCAI 2005-06 | 3d food record | 18-64 | 2313 | 233 | 230-235 | 357 | 65 | Yes ^h^ |
|  | Portugal [42,63] | 8717 | High | National Food, Nutrition and Physical Activity Survey 2015-2016 | 24h recall, FFQ | 18-64 | 17721 | 135 | 135-138 | 418 | 32 | Yes ^h^ |
|  | Serbia [64] | 7618 | Upper-middle | population-based study | 3d 24h recalls, FFQ | 18-49 | 503* | 175 | 168-182 | 302 | 58 | No |
|  | Slovenia [65] | 1718 | High | National nutrition survey 2009 | 24h recall | 18-65 | 410 | 155 | 150-159 | 223 | 69 | Unknown |
|  | Spain [66] | 38469 | High | ENALIA 2 Survey | 2d 24h recalls, FFQ | 18-74 | 1576 | 134 | 127-142 | 328 | 53 | Yes ^m^ |
|  | Turkey [67] | 51828 | Upper-middle | STEPS National survey 2017 | FFQ | ≥15 | 6053 | 128 | 120-144 | 662 | 19 | Yes ^h^ |
| West Europe | Austria [68] | 6985 | High | National nutrition survey 2017 | 2d 24h recalls | 19-65 | 2018 | 207 | 201-213 | 285 | 73 | Yes ^h^ |
|  | Belgium [69] | 8852 | High | the Belgian food consumption survey 2014 | 2d 24h recalls | 18-64 | 1200 | 150 | 144-159 | 379 | 40 | Yes ^h^ |
|  | France [70] | 50233 | High | INCA 3 | 3d 24h recalls | 18-64 | 1600 | 130 | 125-135 | 268 | 48 | Yes ^h^ |
|  | Germany [42,71] | 69439 | High | National nutrition survey 2007 | 2d 24h recalls | 18-64 | 14113 | 95 | 95-96 | 256 | 37 | Yes ^h^ |
|  | Netherlands [72] | 13302 | High | the Dutch National Food Consumption Survey 2012-2016 | 2d 24h recalls | 19-79 | 1047 | 141 | 136-144 | 239 | 58 | Yes ^h^ |
|  | Switzerland [73] | 6620 | High | First National Nutrition Survey menuCH | 2d 24h recalls | 18-75 | 2086 | 196 | 192-199 | 301 | 67 | Yes ^h^ |
| Central America | Guatemala [74] | 8170 | Upper-middle | STEPS National survey 2015 | FFQ | ≥18 | 2036 | 88 | 80-96 | 156 | 56 | No |
|  | Honduras [75] | 4707 | Lower-middle | population-based study | 24h recall, food record | 17-73 | 200* | 0,8 | 0-2 | 127 | 1 | No |
| South America | Argentina [76] | 29370 | High | The Córdoba Obesity and Diet Study (CODIES) | FFQ | ≥18 | 4327 | 263 | 259-266 | 189 | 139 | Yes ^m^ |
|  | Brazil [77] | 141810 | Upper-middle | Health Survey for Sao Paulo 2008-2009 | 2d 24h recalls | ≥18 | 583 | 165 | 145-186 | 142 | 116 | No |
|  | Chile [78] | 13088 | High | National health survey 2009-2010 (ENS) | FFQ | ≥15 | 4958 | 120 | 112-120 | 197 | 111 | Yes ^h^ |
|  | Colombia [79] | 32307 | Upper-middle | STEPS national survey 2010 | FFQ | 15-64 | 2421 | 101 | 95-106 | 120 | 84 | Yes ^h^ |
|  | Ecuador [80] | 10140 | Upper-middle | ENSANUT-ECU 2012 | 24h recall | 19-59 | 10592 | 80 | 72-89 | 64 | 125 | Yes ^h^ |
|  | Paraguay [81] | 4179 | Upper-middle | STEPS National survey 2011 | FFQ | 15-74 | 2538 | 48 | 40-56 | 121 | 40 | Yes ^h^ |
|  | Peru [82] | 19896 | Upper-middle | A randomized cross-sectional household survey | 24h recall | Adults | 240** | 117 | 23-211 | 187 | 161 | No |
|  | Uruguay [83] | 2506 | High | National nutrition survey 2006 | FFQ | 25-64 | 2008 | 120 | 120-120 | 166 | 72 | Yes ^m^ |
| Caribbean countries | Anguilla [84] | 9 | - | STEPS National survey 2016 | FFQ | 18-69 | 1650 | 73 | 66-74 | - | - | Yes ^h^ |
|  | Barbados [85] | 220 | High | STEPS National survey 2007 | FFQ | ≥25 | 1282 | 57 | 50-65 | 200 | 40 | Yes ^h^ |
|  | British Virgin Islands [86] | 20 | High | STEPS National survey 2009 | FFQ | 25-64 | 1094 | 96 | 88-96 | - | - | Yes ^h^ |
|  | Cayman Islands [87] | 40 | High | STEPS National survey 2012 | FFQ | 25-64 | 2105 | 87 | - | - | - | Yes ^h^ |
|  | Dominica [88] | 50 | Upper-middle | STEPS National survey 2008 | FFQ | 15-64 | 1059 | 72 | 64-72 | 252 | 29 | Yes ^h^ |
|  | Grenada [89] | 72 | Upper-middle | STEPS National survey 2010-2011 | FFQ | 25-64 | 1081 | 120 | 104-136 | 111 | 58 | Yes ^h^ |
|  | Haiti [90] | 6041 | Low | population-based study | FFQ | ≥18 | 572 | 98 | 93-103 | 53 | 185 | No |
|  | Jamaica [91] | 1850 | Upper-middle | Population-based study | 12d 24-h recalls, 2 FFQs | ≥25 | 100 | 147 | - | 239 | 61 | No |
|  | Martinique [92] | 264 | - | STEPS National survey 2003-2004 | 24h recall | ≥16 | 1113 | 163 | 154-172 | - | - | Yes ^h^ |
|  | Puerto Rico [93] | 2450 | High | Puerto Rico Health Information National Trends Survey 2009 (HIWTS-PR) | FFQ | ≥18 | 593 | 86 | - | - | - | No |
|  | Saint Kitts and Nevis [94] | 37 | High | STEPS National survey 2008 | FFQ | 25-64 | 1443 | 38 | 30-51 | 73 | 88 | Yes ^h^ |
|  | Saint Lucia [95] | 129 | Upper-middle | STEPS National survey 2012 | FFQ | 25-64 | 1834 | 88 | 80-96 | 93 | 86 | No |
|  | Saint Vincent and the Grenadines [96] | 75 | Upper-middle | STEPS National survey 2013-2014 | FFQ | 18-69 | 3513 | 64 | 56-72 | 122 | 53 | Yes ^h^ |
|  | Trinidad and Tubago [97] | 1011 | High | STEPS National survey 2011 | FFQ | 15-64 | 2631 | 104 | 96-112 | 112 | 93 | Yes ^h^ |
| Northern America | Bermuda [98] | 48 | High | STEPS National survey 2014 | FFQ | ≥18 | 1195 | 144 | 128-160 | 474 | 30 | Yes ^h^ |
|  | Canada [99] | 28159 | High | Canadian Community Health Survey 2007-2014 | 24h recall | ≥19 | 60000 | 226 ^p^ | 221-228 | 301 | 75 | Yes ^h^ |
|  | Mexico [100] | 80390 | Upper-middle | Mexican National Health and Nutrition Survey 2012 | 2d 24h recalls | ≥20 | 3174 | 117 | 117-118 | 153 | 96 | Yes ^h^ |
|  | USA [101] | 241827 | High | National Health and Nutrition Examination Survey 2007-2010 | 24h recall | ≥19 | 11481 | 92 | 86-95 | 316 | 30 | Yes ^h^ |
| North Africa | Algeria [102] | 26391 | Upper-middle | STEPS National survey 2003 | FFQ | 25-64 | 4102 | 160 | - | 455 | 35 | Yes ^h^ |
|  | Egypt [103] | 51934 | Lower-middle | STEPS National survey 2011-2012 | FFQ | 15-65 | 5300 | 88 | 72-96 | 531 | 17 | Yes ^h^ |
|  | Libya [104] | 4059 | Upper-middle | STEPS National survey 2009 | FFQ | 25-64 | 3590 | 96 | 88-96 | - | - | Yes ^h^ |
|  | Morocco [105] | 21998 | Lower-middle | population-based study | 24h recall | ≥17 | 387 | 170 ^u^ | 169-170 | 365 | 46 | No |
|  | Sudan [106] | 19829 | Low | STEPS Subnational survey 2005-2006 | FFQ | 25-64 | 1573 | 112 | - | 230 | 49 | No |
| East Africa | Comoros [107] | 381 | Low | STEPS National survey 2011 | FFQ | 25-64 | 5760 | 28 | 24-33 | - | - | Yes ^h^ |
|  | Eritrea [108] | 3215 | Low | STEPS National survey 2004 | FFQ | 15-64 | 2319 | 56 | - | - |  | Yes ^h^ |
|  | Ethiopia [109] | 47067 | Low | STEPS National survey 2015 | FFQ | 15-69 | 9742 | 48 | 40-56 | 59 | 81 | Yes ^h^ |
|  | Kenya [110] | 22830 | Lower-middle | STEPS National survey 2015 | FFQ | 18-69 | 4484 | 104 | 96-112 | 138 | 75 | Yes ^h^ |
|  | Madagascar [111] | 11622 | Low | STEPS National survey 2005 | FFQ | ≥15 | 5743 | 304 | - | 48 | 632 | Yes ^h^ |
|  | Malawi [112] | 7830 | Low | STEPS National survey 2010 | FFQ | 25-64 | 5177 | 128 | 128-136 | 61 | 211 | Yes ^h^ |
|  | Mozambique [113] | 12441 | Low | STEPS National survey 2005 | FFQ | 25-64 | 3310 | 88 | - | 51 | 173 | Yes ^h^ |
|  | Mauritius [114] | 941 | Upper-middle | Intervention study | FFQ | 19-55 | 178 | 48 | 48-50 | 206 | 23 | No |
|  | Rwanda [115] | 5816 | Low | STEPS National survey 2015 | FFQ | 15-64 | 7232 | 73 | 69-75 | 158 | 81 | Yes ^h^ |
|  | Seychelles [116] | 68 | High | The Seychelles Heart Study 2004 | FFQ | 25-64 | 1255 | 152 | 152-160 | - | - | Yes ^h^ |
|  | Tanzania [117] | 23376,2692 | Low | STEPS National survey 2012 | FFQ | 25-64 | 5680 | 80 | 78-88 | 125 | 64 | Yes ^h^ |
|  | Uganda [118] | 16805 | Low | STEPS National survey 2014 | FFQ | 18-69 | 3967 | 104 | 96-112 | 75 | 139 | Yes ^h^ |
|  | Zambia [119] | 6776 | Lower-middle | STEPS national survey 2017 | FFQ | 18-69 | 3980 | 168 | 160-184 | 71 | 215 | Yes ^h^ |
|  | Zanzibar [120] | 641 | Low | STEPS National survey 2011 | FFQ | 25-64 | 2636 | 56 | 56-56 | - | - | Yes ^h^ |
| Central Africa | Cameroon [121] | 11217 | Lower-middle | population-based, cross-sectional study | FFQ | 21-60 | 571 | 304 | - | 294 | 103 | No |
|  | Central African Republic [122] | 2468 | Low | STEPS Subnational survey 2010 | FFQ | 25-64 | 4029 | 196 | 169-222 | 52 | 503 | Yes ^h^ |
|  | Chad [123] | 5733 | Low | STEPS Subnational survey 2015 | FFQ | 25-64 | 2016 | 77 | - | 21 | 604 | No |
|  | Democratic Republic of the Congo [124] | 32600 | Low | STEPS National survey 2005 | FFQ | ≥15 | 1896 | 34 | 33-36 | 106 | 226 | Yes ^h^ |
|  | Gabon [125] | 925 | Upper-middle | STEPS National survey 2009 | FFQ | 15-64 | 2708 | 25 | 24-29 | 121 | 53 | No |
|  | Sao Tome and Principe [126] | 101 | Upper-middle | STEPS National survey 2009 | FFQ | 25-64 | 2457 | 104 | 93-117 | 44 | 308 | Yes ^h^ |
| West Africa | Benin [127] | 5224 | Low | STEPS National survey 2015 | FFQ | 18-69 | 5036 | 51 | 44-58 | 157 | 61 | Yes ^h^ |
|  | Burkina Faso [128] | 8076 | Low | STEPS National survey 2013 | FFQ | 25-64 | 4417 | 25 | 24-29 | 46 | 138 | Yes ^h^ |
|  | Cape Verde [129] | 319 | Lower-middle | STEPS National survey 2007 | FFQ | 25-64 | 1762 | 112 | 80-144 | 331 | 34 | Yes ^h^ |
|  | Gambia [130] | 878 | Low | STEPS National survey 2010 | FFQ | 25-64 | 4111 | 112 | 96-128 | 58 | 192 | Yes ^h^ |
|  | Ghana [131] | 14304 | Lower-middle | STEPS National survey 2006 | FFQ | 25-64 | 2662 | 128 | - | 117 | 110 | No |
|  | Guinea [132] | 5999 | Low | STEPS Subnational survey 2009 | FFQ | 15-64 | 2491 | 112 | 96-128 | 135 | 83 | No |
|  | Ivory Coast [133] | 10551 | Lower-middle | STEPS National survey 2005 | FFQ | 15-64 | 4530 | 184 | 168-208 | 110 | 168 | No |
|  | Liberia [134] | 2175 | Low | STEPS National survey 2011 | FFQ | 25-64 | 2114 | 80 | 72-88 | 74 | 108 | Yes ^h^ |
|  | Mali [135] | 7055 | Low | STEPS Subnational survey 2007 | FFQ | 25-64 | 2810 | 216 | - | 156 | 139 | No |
|  | Mauritania [136] | 941 | Lower-middle | STEPS National survey 2006 | FFQ | 15-64 | 245 | 152 ^p^ | - | 101 | 151 | No |
|  | Niger [137] | 7750 | Low | STEPS National survey 2007 | FFQ | 15-64 | 2780 | 72 | - | 160 | 45 | Yes ^h^ |
|  | Senegal [138] | 7059 | Low | STEPS National survey 2015 | FFQ | 18-69 | 6306 | 211 | 202-227 | 151 | 196 | Yes ^h^ |
|  | Sierra Leone [139] | 3162 | Low | STEPS National survey 2009 | FFQ | 25-64 | 4997 | 96 | 80-112 | 158 | 61 | Yes ^h^ |
|  | Togo [140] | 3525 | Low | STEPS National survey 2010 | FFQ | 15-64 | 4301 | 72 | 64-80 | 76 | 95 | Yes ^h^ |
| South Africa | Botswana [141] | 1210 | Upper-middle | STEPS National survey 2014 | FFQ | 15-69 | 3519 | 88 | 80-96 | 107 | 82 | Yes ^h^ |
|  | Lesotho [142] | 1168 | Lower-middle | STEPS National survey 2012 | FFQ | 25-64 | 2310 | 168 | 152-184 | 54 | 309 | Yes ^h^ |
|  | Namibia [143] | 1315 | Upper-middle | Namibia Demographic and Health Survey 2013 | 24h recall | 15-64 | 14454 | 102 | - | 95 | 107 | Yes ^h^ |
|  | South Africa [144] | 34410 | Upper-middle | The Cardiovascular Risk in Black South Africans (CRIBSA) Study | 24h recall | 19-64 | 544 | 137 | 127-148 | 119 | 116 | No |
|  | Swaziland [145] | 688 | Lower-middle | STEPS National survey 2014 | FFQ | 15-69 | 3197 | 112 | 104-120 | 58 | 194 | Yes ^h^ |
| Australasia | Australia [146] | 17998 | High | Australian Health Survey 2011-2012 | 2d 24h recalls | 19-70 | 11713 | 127 | 118-135 | 283 | 45 | Yes ^h^ |
|  | New Zealand [147] | 3412 | High | New Zealand Adult Nutrition Survey 2008-2009 | 2d 24h recalls | 15-70 | 3656 | 120 ^l^ | 120-121 | 357 | 34 | Yes ^h^ |
|  | Tokelau [148] | 1 | - | STEPS National survey 2005 | FFQ | 15-64 | 583 | 23 | - | - | - | Yes ^h^ |
| Melanesia | Papua New Guinea [149] | 4058 | Lower-middle | STEPS National survey 2007-2008 | FFQ | 15-64 | 2915 | 64 | 56-64 | - | - | Yes ^h^ |
|  | Solomon islands [150] | 299 | Lower-middle | STEPS National survey 2006 | FFQ | 15-64 | 1902 | 112 | 104-120 | 37 | 450 | Yes ^h^ |
|  | Vanuatu [151] | 143 | Lower-middle | STEPS National survey 2011 | FFQ | 25-64 | 4451 | 248 | 232-272 | 142 | 174 | Yes ^h^ |
| Micronesia | Guam [152] | 98 | High | cross-sectional study | 24h recall | 25-65 | 122 | 216 | 162-270 | - | - | No |
|  | Kiribati [153] | 63 | Lower-middle | STEPS National survey 2004-2006 | FFQ | 15-64 | 1217 | 32 | 32-32 | 155 | 21 | Yes ^h^ |
|  | Marshall Islands [154] | 34 | Upper-middle | STEPS National survey 2002 | FFQ | 15-64 | 2772 | 80 | 64-96 | - | - | Yes ^h^ |
|  | Micronesia [155] | 60 | Lower-middle | STEPS National survey 2006 | FFQ | 25-64 | 1939 | 88 | 80-96 | - | - | No |
|  | Nauru [156] | 6 | Upper-middle | STEPS National survey 2004 | FFQ | 15-64 | 1177 | 96 | 88-104 | - | - | Yes ^h^ |
| Polynesia | American Samoa [157] | 30 | Upper-middle | STEPS National survey 2004 | FFQ | 25-64 | 2038 | 110 | 97-123 | - | - | Yes ^h^ |
|  | Cook Islands [158] | 14 | - | STEPS National survey 2003-2004 | FFQ | 25-64 | 2010 | 120 | 112-136 | - | - | Yes ^h^ |
|  | French Polynesia [159] | 151 | High | STEPS National survey 2010 | FFQ | 18-64 | 3469 | 120 | 112-128 | 140 | 86 | Yes ^h^ |
|  | Hawaii [160] | 775 | - | cross-sectional study | FFQ | ≥18 | 384 | 187 ^pl^ | 187-188 | - | - | Yes ^h^ |
|  | Niue [161] | 1 | - | STEPS National survey 2011-2012 | FFQ | 15-64 | 752 | 72 | - | - | - | Yes ^h^ |
|  | Samoa [162] | 106 | Upper-middle | STEPS National survey 2002 | FFQ | 25-64 | 1817 | 417 | 374-475 | 67 | 683 | Yes ^h^ |
|  | Tonga [163] | 59 | Upper-middle | STEPS National survey 2012 | FFQ | 25-64 | 2429 | 168 | 160-184 | - | - | Yes ^h^ |

*only female participants, ** unknown gender of participants
^u^ legumes excluded, unknown if potatoes are included, ^l^ legumes included, ^pl^ potatoes and legumes included, ^p^ potatoes included
^m^ moderate, ^h^ high

**References**

1. (UNICEF), U.N.C.s.F. *The State of the World’s Children 2015: Executive Summary. Reimagine the Future. Innovation for Every Child.*; New York, 2014.

2. Group, T.W.B. World Bank Country and Lending Groups. Availabe online: <https://datahelpdesk.worldbank.org/knowledgebase/articles/906519-world-bank-country-and-lending-groups> (accessed on 2 March 2019).

3. Kousha, A.; Etemad, K.; Kouhpayezadeh, J.; Abachizadeh, K.; Rafei, A.; Salavati, F.; Darman, M.; Rezanejad, P.; Pariani, A. Iran non-communicable disease risk factors surveillance. National report selected results 2011. **2011**.

4. H.Hussein, A.; Ahmed, I.J.; Huwail, M.J.; Aati, A.S.; Al-Noori, T.A.H.; Al-Siraj, M.A.; Kadhum, D.A.; Ali, M.A.K.; Wahab, F.M.; Al-Alak, M.M., et al. Non-communicable diseases risk factors STEPS survey Iraq 2015. **2015**.

5. Nutrition Surveys (MABAT) Unit. Availabe online: <https://www.health.gov.il/English/MinistryUnits/ICDC/Units/nutrition_surveys/Pages/default.aspx> (accessed on April 2019 ).

6. *Jordan STEPS Survey 2007 Fact Sheet*; World Heath Organization (WHO): 2007.

7. Riley, L.M.; Savin, S.; Guthold, R.; Cowan, M.; AlWotayan, R.; AlDuwairi, Q.; AlWotayan, R.; AlHamad, N.; AlKhalifa, F.; Alawadi, A., et al. EMAN Eastern Mediterranean Approach for Control of Non Communicable Diseases. Survey of Risk Factors for Chronic Non Communicable Diseases. **2015**.

8. Ammar, W.; Hamade, R.; Riedner, G.; Rady, A. WHO STEPwise Approach for Non-Communicable Diseases Risk Factors Surveillance – Lebanon **2017**.

9. Bakri, A.H.; Thani, A.A.A.; Al-Chetachi, W.F.M.; Fadhil, I.; Ratcliffe, C.; Ponte, P.D.; Tagney, M.W. Qatar stepwise report 2012. Chronic disease risk factor surveillance. **2013**.

10. Moradi-Lakeh, M.; El Bcheraoui, C.; Afshin, A.; Daoud, F.; AlMazroa, M.A.; Al Saeedi, M.; Basulaiman, M.; Memish, Z.A.; Al Rabeeah, A.A.; Mokdad, A.H. Diet in Saudi Arabia: findings from a nationally representative survey. *Public Health Nutr* **2017**, *20*, 1075-1081, doi:10.1017/s1368980016003141.

11. Dehghan, M.; Al Hamad, N.; Yusufali, A.; Nusrath, F.; Yusuf, S.; Merchant, A.T. Development of a semi-quantitative food frequency questionnaire for use in United Arab Emirates and Kuwait based on local foods. *Nutrition journal* **2005**, *4*, 18, doi:10.1186/1475-2891-4-18.

12. *Palestine STEPS Survey 2010-2011 Fact Sheet*; World Health Organization: 2011.

13. (WHO), W.H.O. Uzbekistan STEPS Survey 2014 Fact Sheet. **2014**.

14. Li, Y.C.; Jiang, B.; Zhang, M.; Huang, Z.J.; Deng, Q.; Zhou, M.G.; Zhao, Z.P.; Wang, Y.F.; Wang, L.M. Vegetable and Fruit Consumption among Chinese Adults and Associated Factors: A Nationally Representative Study of 170,847 Adults. *Biomedical and environmental sciences : BES* **2017**, *30*, 863-874, doi:10.3967/bes2017.117.

15. Wang, D.H.; Kogashiwa, M.; Mori, N.; Yamashita, S.; Fujii, W.; Ueda, N.; Homma, H.; Suzuki, H.; Masuoka, N. Psychosocial Determinants of Fruit and Vegetable Consumption in a Japanese Population. *Int J Environ Res Public Health* **2016**, *13*, doi:10.3390/ijerph13080786.

16. Choi, M.K.; Bae, Y.J. Vegetable intake is associated with lower Frammingham risk scores in Korean men: Korea National Health and Nutrition Survey 2007-2009. *Nutr Res Pract* **2016**, *10*, 89-98, doi:10.4162/nrp.2016.10.1.89.

17. Hong, S.A.; Kim, M.K. Relationship between fruit and vegetable intake and the risk of metabolic syndrome and its disorders in Korean women according to menopausal status. *Asia Pac J Clin Nutr* **2017**, *26*, 514-523, doi:10.6133/apjcn.042016.03.

18. N.Bolormaa; P.Enkhtuya; D.Otgontuya; B.Ichinkhorloo; Ch.Nyamragchaa; B.Suvd; U.Tserendolgor; B.Sodgerel; J.Batjargal; N.Narantuya, et al. Third national STEPS Survey on the Prevalence of Noncommunicable Disease and Injury Risk Factors-2013. **2014**.

19. *Hong Kong population-based food consumption survey 2005-2007*; The Chinese University of Hong Kong

2010.

20. Wu, S.J.; Pan, W.H.; Yeh, N.H.; Chang, H.Y. Trends in nutrient and dietary intake among adults and the elderly: from NAHSIT 1993-1996 to 2005-2008. *Asia Pac J Clin Nutr* **2011**, *20*, 251-265.

21. Ong, S.K.; Lai, D.T.C.; Wong, J.Y.Y.; Si-Ramlee, K.A.; Razak, L.A.; Kassim, N.; Kamis, Z.; Koh, D. Cross-sectional STEPwise Approach to Surveillance (STEPS) Population Survey of Noncommunicable Diseases (NCDs) and Risk Factors in Brunei Darussalam 2016. **2017**.

22. Rahjeng, E.; Kusumawardhani, N.; Indradjaja, S. Monitoring and Evaluation of the integrated community-based intervention for the prevention of NCD in Depok, West Java, Indonesia. **2010**.

23. Sophal, O.; Raisingsey, P.P.; Vannareth, M.; Sothea, A.; Youttiroung, B.; Sovannara, C.; Linda, P.; Sathia, L. Prevalence of non-communicable disease risk factors in Cambodia. **2010**.

24. Vongvichith, E.; Phounsavath, S.; Vongsamphane, C.; Syhavong, B.; Rassachack, B.; Douangchack, P.; Phongsavath, K.; Douangchak, S.; Chu, V.; Melanie, C., et al. Report on STEPS Survey on Non Communicable Diseases Risk Factors in Vientiane Capital city, Lao PDR. **2010**.

25. Institute, F.a.N.R. 7th National nutrition survey 2008. Food Consumption Survey Component. Individual Food and Nutrient Intake. **2009**.

26. Phu, T.D.; Bac, T.D.; Bao, T.Q.; Toan, H.H.; Diem, N.T.H.; Giang, K.B.; Ha, D.T.P.; Lan, V.H.; Lam, N.T.; Linh, D.H., et al. National survey on the risk factors of non-communicable diseases( STEPS) Vietnam 2015. **2016**.

27. Zainuddin, A.A.; Nazri Jai, A.; Azhana Husna Zainudeen, A.; Baharudin, A.; Mahadir Naidu, B.; Chan, Y.Y.; Siew Man, C.; Chong, Z.L.; Paiwai, F.; Nordin, F., et al. *National Health and Morbidity Survey 2014 : Malaysian Adult Nutrition Survey (MANS) Vol. II : Survey Findings*; 2016; 10.13140/RG.2.1.4039.9769.

28. Kjollesdal, M.; Htet, A.S.; Stigum, H.; Hla, N.Y.; Hlaing, H.H.; Khaine, E.K.; Khaing, W.; Khant, A.K.; Khin, N.O.; Mauk, K.K., et al. Consumption of fruits and vegetables and associations with risk factors for non-communicable diseases in the Yangon region of Myanmar: a cross-sectional study. *BMJ open* **2016**, *6*, e011649, doi:10.1136/bmjopen-2016-011649.

29. *Report of the National Nutrition Survey 2010*; Singapore, 2010.

30. Satheannoppakao, W.; Aekplakorn, W.; Pradipasen, M. Fruit and vegetable consumption and its recommended intake associated with sociodemographic factors: Thailand National Health Examination Survey III. *Public Health Nutr* **2009**, *12*, 2192-2198, doi:10.1017/s1368980009005837.

31. Martins, J.S.; Correira, A.G.; Belo, N.; Santos, H.S.d.; Costa, M.d.C.S.P.d. National survey for  noncommunicable disease risk factors  and injuries using WHO STEPS approach  in Timor-Leste – 2014. **2015**.

32. Kanungsukkasem, U.; Ng, N.; Van Minh, H.; Razzaque, A.; Ashraf, A.; Juvekar, S.; Masud Ahmed, S.; Huu Bich, T. Fruit and vegetable consumption in rural adults population in INDEPTH HDSS sites in Asia. *Global health action* **2009**, *2*, doi:10.3402/gha.v2i0.1988.

33. Dorji, T.; Dukpa, W.; Doma, K.; Pelzom, D.; Gurung, M.S.; Bhatti, L.; Sinha, D.N.; Garg, R.; Agarwal, N. National survey for  noncommunicable disease risk factors  and mental health using WHO STEPS approach in Bhutan – 2014. **2015**.

34. Brahmam, G.N.V.; Laxmaiah, A.; Kumar, R.H.; Arlappa, N.; Balakrishna, N.; Meshram, I.I.; Rao, K.M.; Reddy, C.G.; Bhaskar, V.; Kumar, S., et al. Diet and Nutritional Status of Tribal Population and  Prevalence of Hypertension among Adults  - Report on Second Repeat Survey. **2009**.

35. Mohamed, A.J.; Magtymova, A.; Thaufeeq, U.; Aboobakuru, M.; Nayaz Ahmed, S.M.; Geela Ali, S.A.; Samiya, A.; Krishnan, A.; JS Thakur, R.G.; Riley, L., et al. WHO STEPS survey on risk factors for noncommunicable diseases Maldives, 2011. **2014**.

36. Aryal, K.K.; Neupane, S.; Mehata, S.; Vaidya, A.; Singh, S.; Paulin, F.; Madanlal, R.G.; Riley, L.M.; Cowan, M.; Guthold, R., et al. Non Communicable Diseases Risk Factors: STEPS Survey Nepal 2013. **2014**.

37. Qureshi, H.; Munir, M.A.; Saqib, M.A.N.; Rafique, I.; Khan, S.A.; Cowan, M. Non‐Communicable Diseases Survey ‐ Pakistan 2016. **2016**.

38. Jayawickrama, A.; Mahipala, P.; Somatunga, L.C.; Singh, P.K.; Kumaresan, J.; Somatung, L.C.; Siriwardhan, V.T.S.K.; Mallwarachchi, V.; Sirithung, S.; Rathnayaka, R., et al. Non Communicable Disease Risk Factor Survey Sri Lanka 2015. **2015**.

39. Mammadov, J.; Ibrahimov, F.; Ibrahimova, A.; Jamilova, T. National Survey on Risk Factors for Chronic Noncommunicable Diseases in Azerbaijan. **2011**.

40. Schuler, U.; Heller, S. [Chemotherapy-induced peripheral neuropathy and neuropathic pain]. *Schmerz* **2017**, *31*, 413-425, doi:10.1007/s00482-017-0198-x.

41. (WHO), W.H.O. Belarus STEPS Survey 2016-2017. **2017**.

42. EFSA. The EFSA Comprehensive European Food Consumption Database. Availabe online: <https://www.efsa.europa.eu/en/food-consumption/comprehensive-database> (accessed on 20 June 2019).

43. Ruprich, J.; Dofkova, M.; Rehurkova, I.; Slamenikova, E.; Resova, D. Individual food consumption - the national study SISP04. **2006**.

44. *Georgia STEPS Survey 2016 Fact Sheet.*; 2016.

45. Rodler, I.; Birό, L.; Greiner, E.; Zajkás, G.; Szόrád, I.; Varga, A.; Domonkos, A.; Agoston, H.; Balazs, A.; Mozsary, E., et al. Taplalkozasi vizsgalat Magyarorszagon, 2003-2004. (Dietary survey in Hungary, 2003-2004). **2005**.

46. Guthold, R.; Cowan, M.; Obreja, G.; Cotelea, S.; Cerniciuc, C.; Zatic, T.; Salaru, I.; Obreja, G.; Crudu, P.; Curocichin, G., et al. *Prevalence of non communicable disease risk factors in the Republic of Moldova STEPS 2013*; 2014.

47. Sekula, W.; Nelson, M.; Figurska, K.; Oltarzewski, M.; Weisell, R.; Szponar, L. Comparison between household budget survey and 24-hour recall data in a nationally representative sample of Polish households. *Public Health Nutr* **2005**, *8*, 430-439.

48. Stefler, D.; Pajak, A.; Malyutina, S.; Kubinova, R.; Bobak, M.; Brunner, E.J. Comparison of food and nutrient intakes between cohorts of the HAPIEE and Whitehall II studies. *European Journal of Public Health* **2016**, *26*, 628-634, doi:10.1093/eurpub/ckv216.

49. Pedersen, A.N.; Christensen, T.; Matthiessen, J.; Knudsen, V.K.; Rosenlund-Sørensen, M.; Biltoft-Jensen, A.; Hinsch, H.-J.; Ygil, K.H.; Kørup, K.; Saxholt, E., et al. Danskernes kostvaner 2011-2013. **2015**.

50. Eha, N.; Keiu, N.; Marge, S.; Madli, M.; Liis, N. National Dietary Survey among 11‐74 years old individuals in Estonia. *EFSA Supporting Publications* **2017**, *14*, 1198E, doi:doi:10.2903/sp.efsa.2017.EN-1198.

51. Helldán, A.; Raulio, S.; Kosola, M.; Tapanainen, H.; Ovaskainen, M.-L.; Virtanen, S. Finravinto 2012 -tutkimus The National FINDIET 2012 Survey. **2013**.

52. Þorgeirsdóttir, H.; Valgeirsdóttir, H.; Gunnarsdóttir, I.; Gísladóttir, E.; Gunnarsdóttir, B.E.; Þórsdóttir, I.; Stefánsdóttir, J.; Steingrímsdóttir, L. Hvað borða Íslendingar? Könnun á mataræði Íslendinga 2010-2011 Helstu niðurstöður. **2012**.

53. Albert Flynn, J.W.; Bannon, S.; Browne, F.; Cronin, C.; Cummins, C.; Giltinan, M.; Hennessy, Á.; Hayes, E.; Keyes, L.; Lyons, J., et al. *National Adult Nutrition Survey. Summary Report on Food and Nutrient intakes, Physical Measurements, Physical Activity Patterns and Food Choice Motives*; 2011.

54. Inese, S.; Olga, V.; Gatis, O.; Māris, G. Latvian National Dietary Survey on the general population. *EFSA Supporting Publications* **2017**, *14*, 1307E, doi:doi:10.2903/sp.efsa.2017.EN-1307.

55. Barzda, A.; Baltušytė, R.B.I.; Stukas, R.; Bartkevičiūtė, S. SUAUGUSIŲ IR PAGYVENUSIŲ LIETUVOS GYVENTOJŲ FAKTINĖS MITYBOS IR MITYBOS ĮPROČIŲ TYRIMAS. **2016**.

56. Totland, T.H.; Melnæs, B.K.; Lundberg-Hallén, N.; Helland-Kigen, K.M.; Lund-Blix, N.A.; Myhre, J.B.; Johansen, A.M.W.; Løken, E.B.; Andersen, L.F. Norkost 3 En landsomfattende kostholdsundersøkelse blant menn og kvinner i Norge i alderen 18-70 år, 2010-11. **2012**.

57. Amcoff, E.; Edberg, A.; Barbieri, H.E.; Lindroos, A.K.; Nälsén, C.; Pearson, M.; Lemming, E.W. Livsmedels- och näringsintag bland vuxna i Sverige Resultat från matvaneundersökning utförd 2010–11. **2012**.

58. Bates, B.; Lennox, A.; Prentice, A.; Bates, C.; Swan, G. National Diet and Nutrition Survey. Headline results from Years 1, 2 and 3 (combined) of the Rolling Programme (2008/2009 – 2010/11). **2012**.

59. Majem, L.S.; Alis, J.M.C.; Barba, L.R.; Armengol, G.S.; Quintana, B.R.; Vinas, B.R.; Guitierrez, J.D.; Saban, M.d.C.; Juez, V.A.; Jansana, M.P., et al. *Avalucio de l'estat nutricional de la poblacio d' Andorra. Evolucio dels habits alimentaris i del consum d' aliments a Andorra (2004-2005)*; 2005.

60. Darja, S.; Martina, J.; Sandra, B.; Darko, M.; Davor, I.; Lidija, L.V.; Maja, S. *Croatian National Food Consumption Survey on Adult Population. Croatian Food Agency.*; 2017.

61. Trichopoulou, A.; Papatesta, E.-M.; Klinaki, E.; Martimianaki, G.; Orfanos, P.; Venetis, G.; Valanou, E.; Karathanasis, P.; Nicolaidis, I.; Naska, A. HYDRIA Project: Findings, Conclusions and Proposals for Policy Actions **2016**.

62. Turrini, A.; Leclercq, C.; Arcella, D.; Piccinelli, R.; Sette, S.; Donne, C.L.; Martines, S.; D’Addezio, L. L’indagine nazionale sui consumi alimentari in Italia: INRAN-SCAI 2005-06 **2010**.

63. Carla, L.; Duarte, T.; Andreia, O.; Milton, S.; Sofia, G.; Violeta, A.; Sofia, V.; Elisabete, R.; Sara, R.; Luísa, O., et al. National Food, Nutrition and Physical Activity Survey of the Portuguese general population. *EFSA Supporting Publications* **2017**, *14*, 1341E, doi:doi:10.2903/sp.efsa.2017.EN-1341.

64. Zekovic, M.; Djekic-Ivankovic, M.; Nikolic, M.; Gurinovic, M.; Krajnovic, D.; Glibetic, M. Validity of the Food Frequency Questionnaire Assessing the Folate Intake in Women of Reproductive Age Living in a Country without Food Fortification: Application of the Method of Triads. *Nutrients* **2017**, *9*, doi:10.3390/nu9020128.

65. Blenkuš, M.G.; Gregorič, M.; Tivadar, B.; Koch, V.; Kostanjevec, S.; Turk, V.F.; Žalar, A.; Lavtar, D.; Kuhar, D.; Rozman, U. *PREHRAMBENE NAVADE ODRASLIH PREBIVALCEV SLOVENIJE Z VIDIKA VAROVANJA ZDRAVJA*; 2009.

66. *ENALIA 2 Survey. National Food Survey on adults, the elderly and pregnant women.*; Agencia Española de Seguridad Alimentaria y Nutrición Demométrica: 2016.

67. Kawata, D.; Wu, Z. Regulatable Transgene Expression for Prevention of Chemotherapy-Induced Peripheral Neuropathy. *Mol Ther Methods Clin Dev* **2017**, *6*, 91-101, doi:10.1016/j.omtm.2017.06.004.

68. Rust, P., V. Hasenegger, and J. König. *Österreichischer  Ernährungsbericht 2017.*; 2017.

69. Loes Brocatus, K.D.R., Thérésa Lebacq, Cloë Ost, Eveline Teppers. FOODEX2: VOEDSELCONSUMPTIEGEGEVENS, The Belgian food consumption survey. **2016**.

70. Huneau, M.J.-F.; M. Cyril Feid, H.B.; Blanchemache, S.; Caillavet, F.; Marie-Aline Charles; Christine, M.; Duche, P.; Saillard, P.; Sauvant-Rochat, M.-P.; Walrand, S. Étude individuelle nationale des consommations alimentaires 3 (INCA 3). **2017**.

71. Bell, S.; Braun, G.; Brombach, C.; Eisinger-Watzl, M.; Götz, A.; Hartmann, B.; Heuer, T.; Heyer, A.; Hilbig, A.; Huth, R., et al. *Ergebnisbericht Teil 1 Nationale Verzehrsstudie II*; Max Rubner-Institut; Bundesforschungsinstitut für Ernährung und Lebensmittel: 2008.

72. Rossum, C.T.M.v.; Buurma-Rethans, E.J.M.; Vennemann, F.B.C.; Beukers, M.; Brants, H.A.M.; Boer, E.J.d.; Ocké, M.C. The diet of the Dutch. Results of the first two years of the Dutch National Food Consumption Survey 2012-2016. **2016**.

73. Chatelan, A.; Beer-Borst, S.; Randriamiharisoa, A.; Pasquier, J.; Blanco, J.M.; Siegenthaler, S.; Paccaud, F.; Slimani, N.; Nicolas, G.; Camenzind-Frey, E., et al. Major Differences in Diet across Three Linguistic Regions of Switzerland: Results from the First National Nutrition Survey menuCH. *Nutrients* **2017**, *9*, doi:10.3390/nu9111163.

74. *Guatemala Metropolitan Area - STEPS 2015 Fact sheet.*; 2015.

75. Arps, S. Socioeconomic status and body size among women in Honduran Miskito communities. *Annals of human biology* **2011**, *38*, 508-519, doi:10.3109/03014460.2011.564206.

76. Pou, S.A.; del Pilar Díaz, M.; De La Quintana, A.G.; Forte, C.A.; Aballay, L.R. Identification of dietary patterns in urban population of Argentina: study on diet-obesity relation in population-based prevalence study. *Nutrition Research and Practice* **2016**, *10*, 616-622, doi:10.4162/nrp.2016.10.6.616.

77. Verly, E.; de Carvalho, A.M.; Fisberg, R.M.; Marchioni, D.M.L. Adherence to the food guide for the Brazilian population. *Rev Saude Publ* **2013**, *47*, 1021-1027, doi:10.1590/S0034-8910.2013047004637.

78. Valdivia, C.G.; Margozzini, M.P.; Garrido, M.; Moreira, A.; Araya, M.; Cruz, R.d.l.; Villarroel, L.; Olea, R.; Padilla, O.; Dominguez, A., et al. Encuesta National de Salud ENS Chile 2009-2010. **2010**.

79. Uribe, H.S.; González, W.P.; Flórez, O.A.; Franco, M.H.L.; Martinez, R.G.O.; Sánchez, C.M.H. Factores de riesgo para enfermedades crónicas en Santander, método Stepwise. **2011**.

80. Vance, C.; Chiriboga, D.; Rosero, J.; Villacís, B.; Freire, W.B.; Ramírez-Luzuriaga, M.J.; Belmont, P.; Mendieta, M.J.; Silva-Jaramillo, K.; Romero, N., et al. Encuesta Nacional de Salud y Nutrición Tomo I ENSANUT-ECU 2012. **2014**.

81. Martinez, E.; Giménez, E.; Allende, I.; Cañete, F. Primera Encuesta nacional de factores de riesgo de enfermedades no transmisibles en poblacion general. . **2012**.

82. Penny, M.E.; Meza, K.S.; Creed-Kanashiro, H.M.; Marin, R.M.; Donovan, J. Fruits and vegetables are incorporated into home cuisine in different ways that are relevant to promoting increased consumption. *Matern Child Nutr* **2017**, *13*, doi:10.1111/mcn.12356.

83. Muñoz, M.J.; Galeano, M.F.; Garrido, J.B.; Ferreira, G.R.; Rosa, R. 1ª Encuesta Nacional de Factores de Riesgo de Enfermedades Crónicas No Transmisibles. **2009**.

84. *Anguilla  Stepwise Survey for Noncommunicable Diseases Risk Factors 2016*; Department of Information Technology & E-Government Services (DITES); The Anguilla Statistics Department; The Department of Physical Planning; Caribbean Public Health Agency; Pan American Health Organization

2018.

85. *Barbados STEPS Survey 2007 Fact Sheet.*; 2007.

86. James F. Jekel, D.L.K., Joann G Elmor. STEPS Risk Factor Survey Report. Ministry of Health and Social Development In collaboration with BVI Social Security Board & CAREC/PAHO/WHO. **2010**.

87. Barnes, S.; Watt, C.; Ramoon, E.; Woods-Tatum, B.; McLean, I.; Green, W.; Neil, J.; Martin-Wallace, E.; Sanders, N.; Ebanks, P., et al. WHO STEPS Chronic Disease Risk Factor Survey 2012. **2012**.

88. *Dominica STEPS Survey 2008 Fact Sheet.*; 2008.

89.  *WHO STEPS Chronic Disease Risk Factor Surveillance Grenada.*; Epidemiology Unit, Ministry of Health; Central Statistical Office, Ministry of finance: 2011.

90. Polsinelli, V.B.; Satchidanand, N.; Singh, R.; Holmes, D.; Izzo, J.L., Jr. Hypertension and aging in rural Haiti: results from a preliminary survey. *J Hum Hypertens* **2017**, *31*, 138-144, doi:10.1038/jhh.2016.52.

91. Jackson, M.D.; Walker, S.P.; Younger, N.M.; Bennett, F.I. Use of a food frequency questionnaire to assess diets of Jamaican adults: validation and correlation with biomarkers. *Nutrition journal* **2011**, *10*, 28, doi:10.1186/1475-2891-10-28.

92. Quénel, P.; Blateau, A.; Boudan, V.; Cardoso, T.; Chaud, P.; Malon, A.; Rosine, J.; Merle, S.; Cornely, V.; Flamand, C., et al. Enquête sur la santé et les comportements alimentaires en Martinique (Escal 2003-2004) Résultats du volet "consommations alimentaires et apports nutritionnels". **2008**.

93. Colon-Lopez, V.; Banerjee, G.; Gertz, A.M.; Ortiz, A.P.; Calo, W.; Finney-Rutten, L.J.; Colon-Ramos, U.; Hesse, B.W.; Tortolero, G. Behavioral correlates of fruit and vegetable intake in Puerto Rico: results from the Health Information National Trends Survey. *Puerto Rico health sciences journal* **2013**, *32*, 194-199.

94. Edwards, P.; Williams-Roberts, H.; Sahely, B.; Turner, W.; Fyfield, J. 2008 STEPwise Approach to Chronic  Disease Risk Factor Survey  Report. A baseline for non-communicable disease surveillance in St Kitts. **2008**.

95. *Saint Lucia STEPS Survey 2012 Fact Sheet.*; 2012.

96. *National Health & Nutrition Survey. Non-Communicable Disease Risk Factor Surveillance. Report for St Vincent & the Grenadines.*; Ministry for Health, Wellness and the Environment: 2015.

97. Sundaraneedi, K.; Sharma, K.L.D.; Cumberbatch, A.; Misir, A.; Lewis, Y.; Hinds, A.; Guria, B.K. Panamericans STEPS Chronic non-communicable disease risk factor survey. . **2012**.

98. DeRoza, D.-J. Steps to a  Well Bermuda  2014. **2016**.

99. Colapinto, C.K.; Graham, J.; St-Pierre, S. Trends and correlates of frequency of fruit and vegetable consumption, 2007 to 2014. *Health reports* **2018**, *29*, 9-14.

100. Batis, C.; Aburto, T.C.; Sanchez-Pimienta, T.G.; Pedraza, L.S.; Rivera, J.A. Adherence to Dietary Recommendations for Food Group Intakes Is Low in the Mexican Population. *The Journal of nutrition* **2016**, *146*, 1897s-1906s, doi:10.3945/jn.115.219626.

101. Usual Dietary Intakes: Food Intakes, U.S. Population, 2007-10. Availabe online: <http://epi.grants.cancer.gov/diet/usualintakes/pop/2007-10/index.html> (accessed on May 3, 2018).

102. 2003, S.O.A.t. *Mesure des facteurs de risque des maladies non transmissibles dans deus wilayas pilotes en Algerie 2003*; Algeria, 2005.

103. Ministry of Health and Population, W.c.o.i.E. Egypt STEPS Survey 2011-12 fact sheet. **2012**.

104. (WHO), W.H.O. Libya STEPS Survey 2009 fact sheet. **2010**.

105. Bidi, A.; El Bouhali, B.; Nasri, I.; Eddouks, M. Analysis of food intake profile among women from the oasis of southeastern Morocco. *Eating Behaviors* **2015**, *19*, 90-93, doi:<https://doi.org/10.1016/j.eatbeh.2015.07.001>.

106. (WHO), W.H.O. <Khartoum State/Sudan> STEPS survey 2005-2006 fact sheet. **2006**.

107. (WHO), W.H.O. Union des Comores STEPS  2011 Fact Sheet. **2011**.

108. Mebrahtu, G.; Usman, A.; Nyarang'o, P.; Mufunda, J.; Hagos, G.; brat, Y.G.; E-Mikail, M.; Asgedom; Atange, S.J. National Non communicable disease (NCD) risk factor baseline survey (using WHO STEPwise approach) Ministry of Health Eritrea. **2004**.

109. Ethiopia Public Health Institute, F.M.o.H., World Health Organization (WHO). *Ethiopia STEPS report on risk factors for non-communicable diseases and prevalence at selected NCDs 2015*; Addis Ababa, 2016.

110. Amin, A.; Karagu, A.; Abdikamal, A.; Lubna Bhatti, A.K.; Melanie, C.; Kibogong, D.; Makuba, D.; Kiptui, D.; Tauwo, F.; Ogola, E., et al. Kenya STEPwise survey for non communicable diseases risk factors 2015 report. **2015**.

111. Ministere de la sante et du planning familial, W.H.O.W. Enquête sur les Facteurs de Risque des Maladies Non Transmissibles à Madagascar. **2005**.

112. Malawi, M.o.H. Malawi National STEPS Survey for Chronic Non-Communicable Diseases and their Risk Factors. **2010**.

113. (WHO), W.H.O. Mozambique STEPS survey 2005 fact sheet. **2005**.

114. Pem, D.; Bhagwant, S.; Jeewon, R. A Pre and Post Survey to Determine Effectiveness of a Dietitian-Based Nutrition Education Strategy on Fruit and Vegetable Intake and Energy Intake among Adults. *Nutrients* **2016**, *8*, 127, doi:10.3390/nu8030127.

115. Alypio, N.; Karenzi, A.; Nahimana, R.; Muhimpundu, M.A.; Koama, J.B.; Rusanganwa, A.; Mukazayire, M.F.; Agnes, B.; Raghunathan, P.; Tapela, N., et al. Rwanda Non-communicable Diseases Risk Factors Report 2015. **2015**.

116. Bovet, P.; William, J.; Viswanathan, B.; Madeleine, G.; Romain, S.; Yerly, P.; Paccaud, F.; Gabriel, A. *The Seychelles Heart Study 2004:  methods and main findings*; 2007.

117. Mayige, M.; Kagaruki, G.; Ramaiya, K.; Maongezi, S.; Mbatia, J.; Semu, H.; Saguti, G.; Mghamba, J.; Magimba, A.; Nanai, A. *Tanzania STEPS survey report 2012*; 2013.

118. (WHO), W.H.O. Non‐Communicable Disease Risk Factor Baseline Survey Uganda 2014 Report **2014**.

119. Mutale, W.; Chilengi, R.; Bosaomprah, S.; Siyumbwa, N.; Somwe, P.; Malama, K.; Mukanu, M.; Shankalala, P.; Mwela, C. Zambia Steps For Non Communicable Diseases Risk Factors. Zambia Report for 2017. **2017**.

120. Zanzibar, M.o.H. NCD Survey Report. Main findings from the National Non-communicable disease risk factor survey 2011. **2012**.

121. Nkondjock, A.; Bizome, E. Dietary patterns associated with hypertension prevalence in the Cameroon defence forces. *European journal of clinical nutrition* **2010**, *64*, 1014-1021, doi:10.1038/ejcn.2010.109.

122. (WHO), W.H.O. République Centrafricaine (Bangui) Enquête STEPS 2010. **2011**.

123. publique, R.d.T.M.d.l.s. Republique du Tchad (Site de N’Djaména) Enquête STEPS 2008 fact sheet. **2009**.

124. Longo, M.; Beya, E.; Ekwanzala; Vangu, N.; Nahimana, D.; Mbungu, F.; Bieleli, K.M.B.I.; Mupepe, M. *Enquete sur les facteurs de risque des maladies non transmissibles a Kinshasa, capitale de la RD Congo-2005*; Ministère de la Santé, OMS: Kinshasa, 2006.

125. Comlan, P.; Ayenengoue, C.R.; Baye, E.; Ecke, E.; Ezinah, F.; Ngoungou, E.B.; Kendjo, E.; Nzikoko, J.; Mefe, J.P.Z.; Eya'A, P.O. Enquête sur les facteurs de risque des maladies  non transmissibles à Libreville et Owendo. **2009**.

126. (WHO), W.H.O. São Tomé et Principe Enquête STEPS  2008 Fact Sheet. **2008**.

127. Houinato, D.; Amidou, S.; Sonou, C.H.; Avahoun, V.; Donohuede, S.; Goudjo, G.; Toume, C.; Robin, H.; Mizehoun, C.; Zohoun, I.Y. Rapport final de l’enquête pour la surveillance des facteurs de risque des maladies non transmissibles par l'approche ‘’STEPSwise’’ de l'OMS ENQUETE ‘’STEPS 2015’’ au Bénin. **2016**.

128. Bocar, K.; Amédée, D.P.; Djénéba, S.; Isaïe, M.; Odilon, D.J.E.; Robert, Z.L.; Abdoulaye, B.; Georges, M.; Hervé, T.; Jean-Baptiste, K., et al. *Rapport de l'enquete nationale sur la prevalence des principaux facteurs de risques communs aux maladies non transmissibles du Burkina Faso. Enquete STEPS 2013.*; 2014.

129. (WHO), W.H.O. Cape Verde STEPS Survey 2007 Fact Sheet. **2007**.

130. (WHO), W.H.O. The Gambia STEPS Survey 2010 Fact Sheet. **2010**.

131. (WHO), W.H.O. Ghana STEPS survey 2006 fact sheet. **2006**.

132. (WHO), W.H.O. Guinée (Conakry et Basse Guinée) Enquête STEPS 2009. **2010**.

133. Kouamelan, D.; Ekié, N.Z.; Kouassi, A.; Edouard, A.; Honoré, Z.; Christine, O.Y.; Yao, K.; Baptiste, K.B.J.; Kéita, M.; Soltié, C., et al. *Enquete sur les facteurs de risque des maladies non transmissibles. Côte d’Ivoire - 2005.  Régions sanitaires des Lagunes.*; 2005.

134. Wesseh, C.S.; Peter; Clement; Cowan, M.; team, s.m. Liberia Chronic Disease Risk Factor Surveillance. **2011**.

135. (WHO), W.H.O. Mali (subnational) STEPS Survey 2007 fact sheet. **2007**.

136. BA Mohamed Lemine, D.P. *Enquête sur les Maladies non Transmissibles selon l’approche STEPwise de l’OMS : étude de l’HYPERTENSION ARTERIELLE, du DIABETE et des AUTRES FACTEURS DE RISQUE*; 2007.

137. Souleymane, M.; Bagali, S.; Ali, Y.; Ladou, Y.; Salé, M.; Moussa, H.; Garba, K.; Hassane, M.; Youssouf, Y.; Fatima, A., et al. *Mesure des facteurs de risque des maladies non transmissibles Au Niger  (Approche Step"wise" de l’OMS)*; 2008.

138. (WHO), W.H.O. Sénégal Enquête STEPS  2015 fact sheet. **2015**.

139. (WHO), W.H.O. Sierra Leone STEPS Survey 2009 fact sheet. **2010**.

140. Kokou, A.; Séraphin, A.K.; Kossivi, A.; Kossi, A.; Dégnon, A.; Kwami, A.; Abidè, B.; Yawo, D.; Opportune, D.-E.; Kossi, D., et al. Rapport final de l’enquête STEPS Togo 2010. **2012**.

141. Louazani, S.A.; Cowan, M.; Riley, L.; Guthold, R.; Gaborone; Diaw, M.; Doua, K.; Woldemeskel, T.T.; Masole, N.; Aboeng, S., et al. *Chronic disease risk factor surveillance report 2007*; 2007.

142. (WHO), W.H.O. Lesotho STEPS Survey 2012 Fact Sheet. **2012**.

143. Katjivena, B.; Mbeeli, T.; Nangombe, H.; Shaama, E.; Kakili, T.; Simasiku, M.; Tjiramba, B.; Kamwi, L.; Sagarias, J.; Amutenya, T., et al. *Namibia Demographic and Health Survey 2013*; 2014.

144. Steyn, N.P.; Jaffer, N.; Nel, J.; Levitt, N.; Steyn, K.; Lombard, C.; Peer, N. Dietary Intake of the Urban Black Population of Cape Town: The Cardiovascular Risk in Black South Africans (CRIBSA) Study. *Nutrients* **2016**, *8*, 285, doi:10.3390/nu8050285.

145. Ginindza, C.; Myeni, S.; Nhlebela, N.; Shabangu, P.; Dlamini, T.; Hlophe, P.; Lukhele, V.; Fakudze, T.; Mahlalela, N.; Masuku, B., et al. *WHO STEPS Noncommunicable Disease Risk Factor Surveillance Report*; 2014.

146. Statistics, A.B.o. Australian Health Survey: Nutrition First Results – Food and Nutrients, 2011-12. **2014**.

147. Health., U.o.O.a.M.o. *A Focus on Nutrition: Key findings of the 2008/09 New Zealand Adult Nutrition Survey.*; Wellington, 2011.

148. Nelesone, T.; Pryor, J.; Dan, L.; Tavite, S.; Iosefa, T.; Macdonald, N.; Raj, S.; Riley, L.; Hughes, R.; Galea, G., et al. Tokelau NCD Risk Factors STEPS Report. **2007**.

149. Ogle, G.; Ipai, L.; Dan, L.; Riley, L.; Wari, V.; Guthold, R.; Cowan, M.; Stewart, R.; Davison, L.; Busin, S., et al. Papua New Guinea NCD Risk Factors STEPS report. **2014**.

150. Laesango, N.; Roberts, G.; Dan, L.; Paulsen, J.; Riley, L.; Tuni, M.; Watoto, J.; Raj, S.; Pryor, J. Solomon Islands NCD Risk Factors STEPS report. **2010**.

151. Tarivonda, L.; Tokon, W.; Calo, A.; Garae, B.; Rory, J.J.; Taura, B.; Iaruel, J.; Tabi, G.; Woleg, M.; Ilaisa, E., et al. Vanuatu NCD Risk Factors  STEPS report. **2013**.

152. Guerrero, R.T.; Paulino, Y.C.; Novotny, R.; Murphy, S.P. Diet and obesity among Chamorro and Filipino adults on Guam. *Asia Pac J Clin Nutr* **2008**, *17*, 216-222.

153. Iuta, T.; Phongsavan, P.; Dan, L.; Riley, L.; Metai, A.; Raj, S.; Pryor, J.; Naidu, S. Kiribati  NCD Risk Factors  STEPS report. **2009**.

154. Langidrik, J.R.; Alfred, J.M.; Briand, K.; Nathan, A.; McIntyre, R.; Banda, D.; Nathan, F.; Wase, K.; Lalita, P.; Elbourne, S., et al. Republic of the Marshall Islands NCD Risk Factors STEPS Report 2002. **2007**.

155. Samo, M.; Roberts, G.; Dan, L.; Marar, J.; Riley, L.; Shomour, M.; Raj, S.; Lippwe, K.; Fred, D.; Elymore, A., et al. Federated States of  Micronesia (Chuuk)  NCD Risk Factors  STEPS report. **2012**.

156. Keke, K.; Phongsavan, P.; Dan, L.; Bacigalupo, M.; Smith, B.; Thoma, R.; Riley, L.; Waidubu, G.; Galea, G.; Pryor, J., et al. Nauru NCD Risk Factors STEPS Report. **2007**.

157. Maga, A.a.; Courten, M.d.; Dan, L.; Uele, F.; Macdonald, N.; Lili'o, L.a.; Prior, J.; Riley, L.; Raj, S.; Hughes, R., et al. American Samoa NCD Risk Factors STEPS Report. **2007**.

158. Tairea, K.; Phongsavan, P.; Dan, L.; Fariu, R.; Riley, L.; Avare, T.; Raj, S.; Daniel, R.; Short, N. Cook Islands  NCD Risk Factors STEPS report. **2011**.

159. Riley, L.; Guthold, R.; Cowan, M.; Dan, L.; Tumahai, T.; Marghem, D.; Pujo, J.-M.; Brugiroux, M.-F.; Mallet, H.-P.; Mouchard-Rachet, A., et al. Enquête Santé 2010 en Polynésie française. **2012**.

160. Ollberding, N.J.; Nigg, C.R.; Geller, K.S.; Horwath, C.C.; Motl, R.W.; Dishman, R.K. Food outlet accessibility and fruit and vegetable consumption. *American journal of health promotion : AJHP* **2012**, *26*, 366-370, doi:10.4278/ajhp.101215-ARB-401.

161. Nosa, M.; Roberts, G.; Raj, S.; Bell, C. Niue NCD Risk Factors STEPS report. **2013**.

162. (WHO), W.H.O. Samoa STEPS Survey Fact Sheet. **2002**.

163. Tekiteki, C.L.; Phongsavan, P.; Dan, L.; Riley, L.; Cowan, M.; Kaho, F.; ‘Akau’ola, S.; Raj, S.; Fusimalohi, L. Kingdom of Tonga NCD Risk Factors STEPS Report (2014). **2012**.
